# Comprehensive overview of microRNA function in rheumatoid arthritis

**DOI:** 10.1038/s41413-023-00244-1

**Published:** 2023-01-24

**Authors:** Xiaole Peng, Qing Wang, Wenming Li, Gaoran Ge, Jiachen Peng, Yaozeng Xu, Huilin Yang, Jiaxiang Bai, Dechun Geng

**Affiliations:** 1 Department of Orthopedics, The First Affiliated Hospital of Soochow University, 188 Shizi Street, Suzhou, 215006 Jiangsu P. R. China; 2 Department of Orthopedics, Affiliated Hospital of Zunyi Medical University, 563000 Zunyi, P. R. China

**Keywords:** Bone, Pathogenesis

## Abstract

MicroRNAs (miRNAs), a class of endogenous single-stranded short noncoding RNAs, have emerged as vital epigenetic regulators of both pathological and physiological processes in animals. They direct fundamental cellular pathways and processes by fine-tuning the expression of multiple genes at the posttranscriptional level. Growing evidence suggests that miRNAs are implicated in the onset and development of rheumatoid arthritis (RA). RA is a chronic inflammatory disease that mainly affects synovial joints. This common autoimmune disorder is characterized by a complex and multifaceted pathogenesis, and its morbidity, disability and mortality rates remain consistently high. More in-depth insights into the underlying mechanisms of RA are required to address unmet clinical needs and optimize treatment. Herein, we comprehensively review the deregulated miRNAs and impaired cellular functions in RA to shed light on several aspects of RA pathogenesis, with a focus on excessive inflammation, synovial hyperplasia and progressive joint damage. This review also provides promising targets for innovative therapies of RA. In addition, we discuss the regulatory roles and clinical potential of extracellular miRNAs in RA, highlighting their prospective applications as diagnostic and predictive biomarkers.

## Introduction

Rheumatoid arthritis (RA) is a chronic, inflammatory, autoimmune disorder characterized by progressive and irreversible joint damage as a consequence of sustained synovitis. Patients with RA typically experience pain, swelling and stiffness in multiple joints bilaterally. Extra-articular organs and systems may also be involved as RA proceeds. Cardiovascular disease is a significant and potentially lethal comorbidity, and the risk of its occurrence is intimately related to RA disease activity.^1^ RA is a global health issue, and the prevalence of RA is ~240 per 100 000 individuals. Furthermore, RA prevalence is increasing with the rapidly expanding aging population throughout the world.^2^ The etiology and pathogenesis of RA, involving genetic predisposition, environmental triggers and epigenetic modifications, are highly complex and remain to be elucidated.^3,4^ Synovial hyperplasia, persistent inflammation, articular cartilage degradation and bone erosion are generally considered the cardinal hallmarks of RA pathology.^5,6^

In the past two decades, considerable insights into RA pathophysiology have fostered the development of novel therapeutics in the forms of both biologic and small molecule inhibitors.^7^ Despite this progress, a notable percentage of RA patients still suffer from progressive disability, loss of function, decreased quality of life and diminished life expectancy due to inadequate treatment. Thus, a deeper understanding of the cellular and molecular mechanisms underlying synovial hyperplasia, chronic inflammation and tissue destruction during RA is required to achieve improvement in clinical outcomes.

**Fig. 1:**
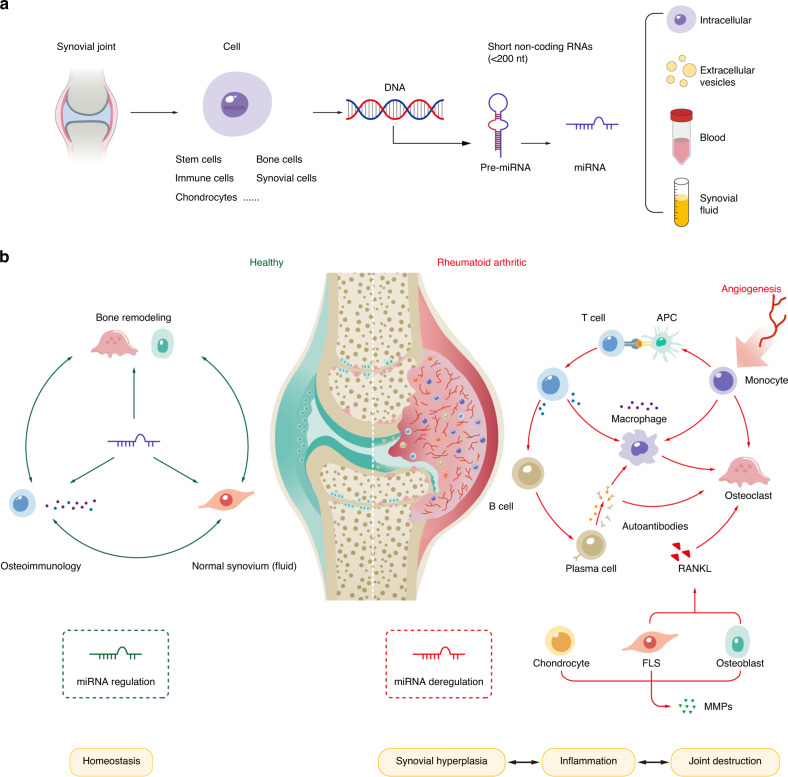
Schematic diagram of miRNAs in normal development, homeostasis and RA progression. **a** In normal synovial joints, many cell types serve as major sources of short noncoding RNAs, including miRNAs, which are transcribed from DNA. Precursor miRNAs are cleaved and processed into miRNAs, which further function within parental cells and neighboring functional cells and can be secreted into biological fluids such as blood and joint fluid as free molecules or extracellular vesicles. **b** In normal conditions in synovial joints, miRNAs are mainly produced by a variety of cells involved in the composition of bone remodeling, osteoimmunology and synovial systems. MiRNAs procedurally regulate well-organized bone remodeling, appropriate levels of immune responses and the secretion of normal synovial fluid. Conversely, persistent synovial hyperplasia, progressive inflammation, and subsequent destruction of affected joints are prominent characteristics of RA. The development, differentiation and homeostasis of diverse cell populations in the inflamed synovial compartment are dysregulated as a result of miRNA deregulation. Interactions between these abnormal cell types with disrupted function ultimately contribute to RA pathogenesis. miRNA microRNA, APC antigen-presenting cell, RANKL receptor activator of NF-κB ligand, FLS fibroblast-like synoviocyte, MMP matrix metalloproteinase

MicroRNAs (miRNAs), a class of short noncoding RNA molecules, are posttranscriptional regulators of gene expression. The first known miRNA, *lin-4*, was recognized in *Caenorhabditis elegans* in 1993.^8^ For the past few years, the field of miRNA biology has expanded considerably. MiRNAs have been shown to play essential roles in the developmental timing of stage-specific cell lineages and control multiple biological processes related to development, differentiation, growth, and metabolism.^9^ The widely observed deregulation of miRNAs in diverse diseases, including cancer,^10^ cardiovascular disorders and inflammatory diseases,^11,12^ has rendered miRNAs attractive candidate targets. In addition, the role of miRNAs in the development of RA has recently attracted attention, as a growing body of evidence has revealed the aberrant expression of numerous miRNAs in clinical samples and experimental models of RA. Studying deregulated miRNAs in RA will provide novel mechanistic and therapeutic insights into the progression of the disease (Fig. 1).

## Cellular landscape in RA pathology

RA is a systemic disease characterized by a complex pathogenesis involving interactions between various cell types located in synovial compartments and peripheral blood, rather than resulting from a single pathogenic factor.^5,6^ These cell populations, comprising fibroblast-like synoviocytes (FLSs), innate and adaptive immune cells, and bone-related cells, change in number, status, and behavior in response to the dynamic microenvironment, which perturbs cytokine secretion, intracellular signaling networks and homeostasis and consequently leads to corresponding pathology. Multiple dysfunctional cell types and the crosstalk between these pathogenic cells collectively contribute to the onset, progression and perpetuation of RA.

### Fibroblast-like synoviocytes (FLSs)

FLSs are highly specialized mesenchymal-derived cells that are a prominent component of the synovial lining layer of diarthrodial joints. Under normal physiological conditions, FLSs express components of the extracellular matrix (ECM) and synovial fluid to lubricate and nourish cartilage surfaces, thereby maintaining the homeostasis of joints. However, accumulating studies have identified FLSs as key players in many pathogenic events in the RA synovium. In pathological conditions, such as RA, FLSs increase rapidly in number and are redistributed in the synovium and joints, exhibiting heterogeneity across different locations within the synovium and across different joints.^13,14^ FLSs in RA display unique aggressive behavior that arises from their reduced rate of apoptosis; deregulated proliferation, migration, and invasion; and improved ability to secrete inflammatory mediators and matrix metalloproteinases (MMPs) into the synovial fluid.^13,15,16^ Researchers have detected constitutive overexpression of the tumor suppressor p53 and mutations in the gene encoding p53 in FLSs from RA patients. Interestingly, these mutations are similar or identical to those found in certain tumors but are not found in skin fibroblasts from the same patients or in FLSs from osteoarthritis (OA) patients.^17^

### Immune cells

Immune dysfunction is undoubtedly a key factor in RA pathogenesis. A variety of immune cells are implicated in and mediate autoimmune inflammation in RA, among which CD4^+^ T lymphocytes (T cells) and monocyte-macrophage lineage cells are acknowledged as the two most significant cellular components.^18^

#### Adaptive immune cells

The significant number of infiltrating T cells, primarily CD4^+^ T cells, in the inflammatory synovial milieu and circulation of patients with RA implies that T cells are one of the major players in RA pathology. CD4^+^ T cells include helper T cells (Th cells), regulatory T cells (Tregs) and follicular helper T cells (Tfh cells). The specific CD4^+^ T-cell subpopulations and their secreted cytokines play distinct and essential roles in the pathogenesis of RA and experimentally induced arthritis.^19^ Pathogenic Th17 cells secreting proinflammatory cytokines, such as interleukin (IL)-17A, IL-17F, and IL-22, are regarded as the predominant positive regulators of the immune response in RA.^5^ Attention has also been increasingly focused on the imbalance of Th17 cells/Tregs. Forkhead box P3 (Foxp3)-positive Tregs, a subset with low proliferative capacity, can secrete cytokines such as IL-4, IL-10 and TGF-β to exert immunosuppressive effects, thereby managing immune homeostasis and inducing immune tolerance. Treg differentiation was reported to be significantly inhibited in patients with RA.^20^ However, others have noted high levels of proliferating Tregs in inflamed joints of RA patients, suggesting that expansion of Tregs is induced by autoimmune inflammatory environments.^21^ Despite the controversy concerning Treg frequencies, it is clear that Tregs become less functional or even pathogenic in the setting of RA. Factors controlling Th17 cell and Treg plasticity may be attractive targets for RA immunotherapy.

The role of B lymphocytes (B cells) in RA pathogenesis goes beyond autoantibody production, antigen presentation and cytokine release. Toll-like receptors (TLRs) drive the differentiation of self-reactive B cells into plasma cells that synthesize autoantibodies.^22^ The best known RA-associated autoantibodies are rheumatoid factor (RF) and anti-citrullinated protein antibody (ACPA). In particular, ACPA has the most pronounced predictive value for the onset of RA in symptomatic, at-risk patients.^23^

#### Innate immune cells

Innate immunity is the first line of defense against the invasion of pathogenic microorganisms. Monocytes and macrophages, as the most important components of the innate immune system, are also key effectors of synovitis in RA. Synovial tissue-resident macrophages in the steady state reside in healthy synovium and perform homeostatic functions but attain an activated phenotype under inflammatory conditions.^24^ Despite having controversial cellular origins,^25^ these tissue-resident macrophages are deemed to self-renew locally throughout adulthood without continuous repopulation from circulating monocytes.^26^ In the context of RA, in addition to activated tissue-resident macrophages, massive bone marrow-derived monocytes accumulate in the blood and consistently migrate into the inflamed joints, where they can differentiate into macrophages to replenish the synovial macrophage pool and thus induce inflammatory reactions.^27,28^ However, infiltrating macrophages are difficult to distinguish from tissue-resident macrophages in diseased human tissues due to the absence of specific markers. Notably, macrophages display remarkable plasticity and heterogeneity, with dynamic phenotypes and distinct functions that are modulated by the surrounding microenvironmental signals.^29^ For simplicity, researchers have constructed a polarization model that classifies macrophage phenotypes on a continuum in which classically activated or inflammatory macrophages (M1) and alternatively activated or reparative macrophages (M2) represent the two opposite extremes.^30^ While disruption of the M1/M2 ratio, which is usually inclined toward M1 polarization, is considered to be one of the pathogenic factors during RA,^31^ the molecular mechanisms that govern M1/M2 polarization remain incompletely defined.

In addition to the monocyte-macrophage lineage, several other innate effector cells, such as dendritic cells (DCs) and neutrophils, are also critical players in the RA process. Indeed, the large amounts of neutrophils, which mainly reside in synovial fluid, contribute to sustained synovitis in RA via the synthesis of proteases, prostaglandins and reactive oxygen intermediates.^32^ DCs might be involved in the promotion of tolerance by mediating the generation and maintenance of Tregs as well as T-cell unresponsiveness.^33^ Conversely, DCs exhibit antigen presentation capacity and can thus foster the priming and/or effector differentiation of self-reactive T cells. The majority of antigen-presenting cells (APCs) in the inflamed RA synovium are fully differentiated DCs expressing high levels of class I and II major histocompatibility complex (MHC) and several T-cell costimulatory molecules.^34^ In summary, the role of DCs in the pathogenesis of RA is complex and dichotomous, and DCs may induce either tolerance or autoimmunity based on the integrated signals received in the microenvironment of synovial joints.

### Bone-related cells

#### Osteoclasts

RA is an independent risk factor for generalized osteopenia and osteoporosis. Osteoclasts, long considered the only effector cells in vivo that participate in bone remodeling by resorbing bone matrix, have emerged as an important cell type in the process of bone erosion in RA. Osteoclasts are multinuclear giant cells that are differentiated from monocyte-macrophage lineage precursor cells derived from bone marrow hematopoietic cells.^35^ The bone matrix can be degraded through matrix enzymes synthesized and secreted by mature osteoclasts, including tartrate-resistant acid phosphatase type 5 (TRAcP), cathepsin K and MMP9. Classically, in the presence of macrophage colony-stimulating factor (M-CSF), the receptor activator of nuclear factor kappa B (NF-κB) ligand (RANKL) pathway and costimulatory signaling pathways synergistically trigger osteoclastogenesis.^36,37^ In the pathogenesis of RA, osteoclasts colonize the interface between the inflamed synovium and the periarticular bone surface. The abundant cytokines, particularly TNF-α, IL-1, IL-6 and IL-17, autoantibodies and RANKL in the inflammatory synovial milieu collectively accelerate bone resorption by fostering osteoclast differentiation and activation.^35,38,39^

#### Osteoblasts

Despite considerable attention being paid to osteoclasts in RA inflamed joints, increasing evidence has suggested that the synovial microenvironment in RA has the potential to induce not only osteoclast differentiation but also osteogenic inhibition. The disruption of bone homeostasis is the result of bone metabolism disorder, in terms of not only excessive bone resorption mediated by osteoclasts but also insufficient bone formation mediated by osteoblasts.^35,40^ Osteoblasts, which originate from mesenchymal stem cells (MSCs) in the bone marrow stroma, are indispensable for the synthesis of the bone matrix and its subsequent mineralization. Impaired osteoblast differentiation and function in RA limit the capacity of bone remodeling and result in severe and ongoing arthritic bone loss at erosion sites.^40^

#### Chondrocytes

Irreversible destruction of articular cartilage is one of the core pathological characteristics of RA.^5^ Articular cartilage is a complex tissue that is composed of chondrocytes and cartilage matrix produced by chondrocytes. The cartilage matrix is composed of collagen and proteoglycan aggregates, which are the structural components of joint tissue and provide biomechanical strength.^41^ Because there is no blood supply in articular cartilage tissue, its nutrition comes entirely from synovial fluid secreted by normal synovium. Therefore, the normal synovial fluid secreted by healthy synovium is important for the maintenance and function of physiological articular cartilage. In the pathogenesis of RA, due to abnormal synovial fluid secretion and invading synovial pannus formation, abundant proinflammatory cytokines are produced in the synovium, which increases MMP secretion by chondrocytes.^42^ Driven by synovial inflammatory microenvironments, anomalous ECM metabolism in coordination with deranged chondrocyte development and function leads to disruption of cartilage homeostasis.^43^

### Crosstalk between pathogenic cell types

**Fig. 2:**
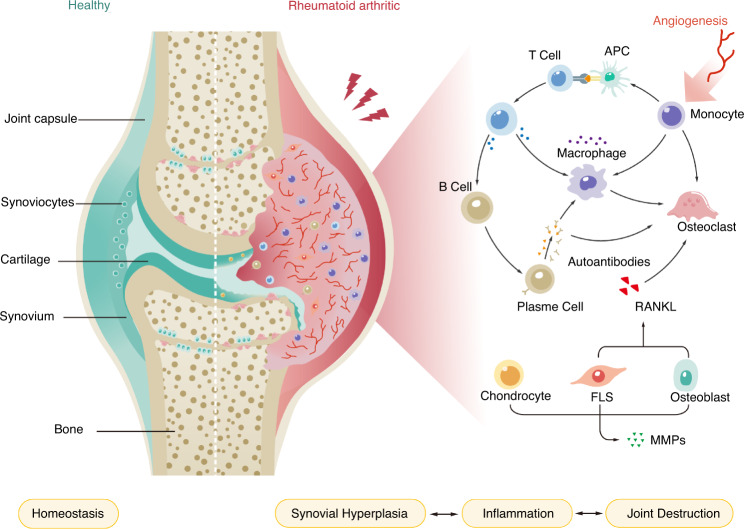
Intercellular crosstalk in RA pathology. APC antigen-presenting cell, RANKL receptor activator of NF-κB ligand, FLS fibroblast-like synoviocyte, MMP matrix metalloproteinase

In the context of RA, starting with an early breach of immune tolerance, dysfunctional leukocytes in circulation massively infiltrate the synovial tissue, accompanied by uncontrolled accumulation of resident synoviocytes. This ongoing process results in the overproduction of proinflammatory mediators and therefore the chronicity of synovitis.^6,44^ Activated synoviocytes also secrete various proangiogenic factors to induce angiogenesis in RA.^45^ Sprouting angiogenesis and pathological vascular remodeling support the increased transendothelial infiltration of blood-derived leukocytes in the inflamed joints. Antigen-specific T-cell/APC interactions result in the proliferation and differentiation of T cells and the secretion of abundant lymphokines to trigger B-cell and macrophage activation. B cells differentiate into plasma cells that mediate autoantibody release and immune complex formation. These immune complexes subsequently induce macrophages to produce proinflammatory cytokines and facilitate osteoclast-mediated bone resorption.^46,47^ Intriguingly, expansion of RA-FLSs promotes hyperplastic transformation of the synovial lining layer and its transformation into an invasive pannus nourished by neovascularization. The pannus exhibits tumor-like behaviors that mediate structural joint destruction through invasion of cartilage and periarticular bone at the cartilage-bone interface.^48,49^ RANKL expressed by FLSs and osteoblasts is also associated with bone erosion because it fosters osteoclastogenesis. Furthermore, in the presence of inflammatory factors, FLSs, chondrocytes and osteoblasts can secrete MMPs to degrade articular cartilage. In light of the above pathological features, cellular interaction-dependent core immune dysfunction and altered tissue homeostasis seem to underlie the disease (Fig. 2).

## Basic biology of MiRNAs

### MiRNA biogenesis and function

**Fig. 3:**
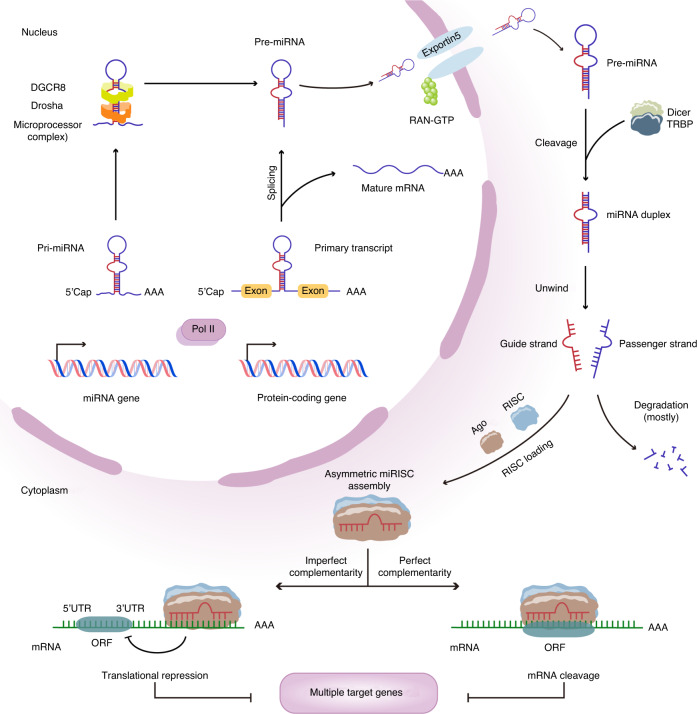
Schematic overview of the biogenesis and mechanism of action of miRNAs. MiRNAs encoded in introns of protein-coding genes and intergenic areas are initially transcribed by RNA Pol II in the nucleus. In the canonical pathway (left), pri-miRNAs are recognized and processed by the microprocessor complex, consisting of two RNase III enzymes, Drosha and DGCR8, releasing hairpin-structured pre-miRNAs. In the noncanonical pathway (right), however, pre-miRNA hairpins are generated from introns that undergo splicing and debranching, bypassing the step of Drosha cleavage. The nuclear export of pre-miRNAs is mediated by Exportin-5 in complex with Ran-GTP. Cytoplasmic pre-miRNAs are further cleaved by the endonuclease Dicer, aided by TRBP, to yield ~22-nucleotide miRNA duplexes. One strand of the duplex (the guide strand) is retained as mature miRNA and incorporated into RISC (containing AGO proteins), whereas the other strand (the passenger strand) is degraded. Mature miRNAs fine-tune gene expression by guiding RISC interaction with complementary sites of target mRNAs. The mechanisms of miRNA-mediated gene silencing, including accelerated cleavage and restricted translation of mRNAs, seem to largely depend on the degree of complementarity between seed sequences of miRNAs and the 3’ UTRs of target mRNAs. miRNA microRNA, Pol II RNA polymerase II, pri-miRNA primary transcript, DGCR8 DiGeorge syndrome critical region gene 8, pre-miRNA precursor miRNA, TRBP TAR RNA-binding protein, RISC RNA-induced silencing complex, AGO Argonaute, 3′ UTR 3′ untranslated region

MiRNAs are a group of evolutionarily conserved endogenous noncoding RNA molecules 22 nucleotides (nt) in length. These small RNAs have tightly regulated expression patterns and serve as posttranscriptional regulators of gene expression.^50^ Most miRNAs are expressed in a temporal and tissue-specific manner, meaning that miRNAs are differentially expressed in various stages of organism development and that the expression of identical miRNAs may vary in different organs and tissues.^51^ Genes encoding miRNAs in mammals are generally localized within the introns of protein-coding genes. However, there are also some miRNAs that originate from intergenic regions and act as independent transcription units.^52,53^ The process of miRNA biogenesis involves multiple steps (Fig. 3), comprising a canonical pathway dependent on Drosha processing and a noncanonical pathway in which pre-miRNAs can be alternatively generated via spliceosome machinery.^50,53–55^ Mature miRNAs mediate the inhibition of gene expression at the posttranscriptional level, which is associated with cellular function maintenance. The efficiency and mode of gene silencing depend on whether the miRNA has sufficient complementarity to the target mRNA.^9,56^ A perfect match between an miRNA and the 3′ UTR of its target leads to irreversible mRNA decay, while imperfect complementarity promotes the repression of translation at the initiation or elongation stages.^57,58^

More than 60% of protein-coding genes in mammals seem to have conserved miRNA target sites.^59^ Each individual gene can be simultaneously targeted by multiple miRNAs, while a single miRNA species has the capacity to interact with a broad range of target genes.^9,53^ Thus, functional miRNAs constitute a huge and complex regulatory network involving extensive signaling cascades, thereby playing fundamental roles in diverse biological processes, including organism development, metabolism, homeostasis, and cellular behaviors such as proliferation, differentiation, migration, and apoptosis. Disturbed expression and functions of these miRNAs under specific circumstances may bring about pathological phenotypes and/or anomalous biological outcomes.^9^

### Extracellular miRNAs and cell-to-cell communication

Numerous miRNAs can be encapsulated within cell-derived membranous structures called exosomes that can be transferred to neighboring or distant cells, thereby facilitating crosstalk between donor cells and recipient cells.^60,61^ Exosomes are the most well-studied class of extracellular vesicles (EVs) and have an average diameter of 50–150 nm.^62^ It has been well established that the transfer of metabolites and biological information between different cell types via exosomal miRNAs is crucial for the coordination of various physiological and pathological processes.^63,64^

The generation, trafficking, and function of exosomes depend on their different intercellular origins and can be modified by other pathological signals and stimuli that the cell may receive.^62^ In essence, exosome biogenesis starts with the formation of the early endosome. Subsequently, intraluminal vesicles (ILVs) are formed and accumulate through inward budding of the endosomal membrane during the maturation of multivesicular endosomes (MVEs). Finally, ILVs are secreted into the extracellular space upon fusion of MVEs with the plasma membrane.^60,65^ ILVs contain and carry complex biomolecules, including various types of proteins, lipids, and nucleic acids, such as DNA sequences, mRNAs, miRNAs, and other noncoding RNAs. Sorting and clustering of these exosomal cargoes into exosomes are largely dependent on particular endosomal sorting machineries.^62^

Interestingly, the enrichment of exosomal miRNAs is different for each cell type. To date, the precise mechanisms involved in the specific loading of miRNAs into exosomes remain elusive. Several potential pathways have been proposed to perform exosomal miRNA sorting. The presence of specific sequence motifs in certain miRNAs seems to determine whether they are incorporated into exosomes or retained in the cell in a cell-specific fashion.^66^ Heterogeneous nuclear ribonucleoprotein A2B1 (hnRNPA2B1) in exosomes critically regulates this process by directly binding to these exosomal miRNAs. Specifically, preferential sumoylation of hnRNPA2B1 fosters its recognition of specific short motifs in miRNAs and guides their localization into exosomes.^67^ hnRNPA2B1 was the first protein identified to be implicated in the selectivity of miRNA loading. Likewise, several RNA-binding proteins that control the export of miRNAs into exosomes have been described. These include Syncrip,^68,69^ major vault protein (MVP),^70^ HuR,^71^ and Y-box protein I (YBX1),^72,73^ each of which has multiple potential miRNA targets. More recently, another two RNA-binding proteins, Alyref and Fus, were discovered to strongly recognize the CGGGAG motif and target miRNAs to exosomes.^66^ The 3′-end of sequence-dependent miRNAs is a potential sorting signal for governing the export of miRNAs as well. 3′-Adenylated miRNAs are preferentially retained in the cell, whereas 3′-uridylated miRNAs are highly exosome-enriched.^74^ In addition, the correlation between miRISC and MVEs suggests another miRNA sorting machinery.^75^ KRAS activity can modulate the colocalization of Ago2, a key component of miRISC, with MVEs. The KRAS–MEK signaling pathway may control the loading of specific exosomal miRNAs through Ago2 phosphorylation.^76^ Deficiency of AGO2 has been observed to restrict the enrichment of some preferentially exported miRNAs, such as miR-451, miR-150, and miR-142-3p, in exosomes derived from multiple cell types.^77^

In summary, a better understanding of exosome biogenesis and how miRNAs are differentially sorted into exosomes may be useful for the artificial packing of selected miRNAs into exosomes, aiding the development of engineered exosomes for gene therapy.

## Deregulated MiRNAs in RA

With the advances in several detection technologies, including microarrays and next-generation sequencing (NGS), aberrant changes in specific and global miRNA levels have been detected in many pathogenic cell types in RA.^78^ Notably, as the expression and function of miRNAs are cell type-specific,^79^ their overall contribution to RA progression—that is, whether they exacerbate or slow progression—is not entirely clear. Given the acknowledged role of miRNAs in fine-tuning gene expression, studying their complex effects on cellular function is bound to prove important for elucidating RA pathogenesis (Fig. 1).

### MiRNA-mediated dysfunction in FLSs

**Fig. 4:**
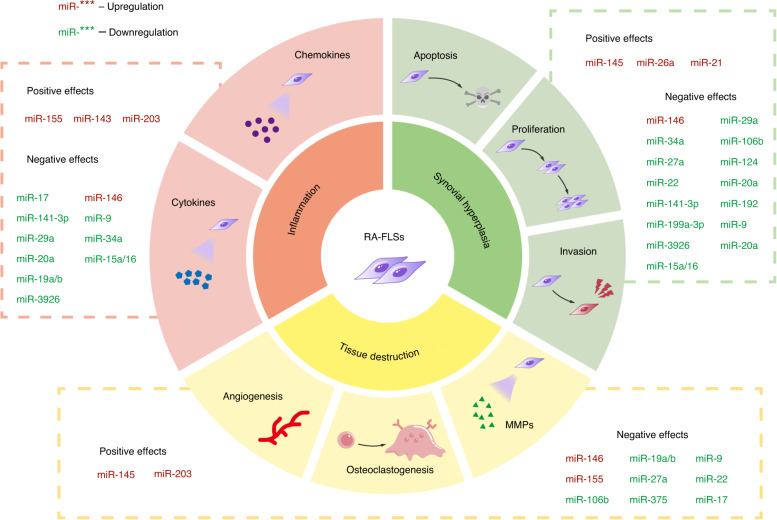
Deregulated miRNAs in RA-FLSs. Upregulation (red font) or downregulation (green font) of several miRNAs is observed in RA-FLSs. These deregulated miRNAs exert positive or negative regulation on the aggressive phenotypes, inflammatory mediator production, and potential to disrupt synovial joint tissues of RA-FLSs. miRNA microRNA, RA rheumatoid arthritis, FLS fibroblast-like synoviocyte, MMP matrix metalloproteinase

Dysregulation of several miRNAs is responsible for the aberrant biological behaviors of FLSs in RA (Fig. 4). Various intracellular pathways are related to the altered expression of miRNAs, and the most significantly implicated pathways are the Wnt, NF-κB, Janus kinase/signal transducer and activator of transcription (JAK/STAT) and TLR pathways. The first two miRNAs found to be differentially expressed in RA-FLSs were miR-155 and miR-146a.^80^ The expression of miR-155 is prominently upregulated in RA-FLSs when they are stimulated with tumor necrosis factor (TNF)-α. In addition to its widely reported proinflammatory role in immune cells, miR-155 also upregulates the secretion of inflammatory factors from RA-FLSs.^81,82^ Of interest, miR-155 is assumed to protect against acquisition of a destructive phenotype by RA-FLSs by repressing the expression of MMP3 and MMP1.^80,83^ In this regard, further research is warranted to dissect the precise role of miR-155 in RA-FLSs and identify its direct target genes and pathways. MiR-146a, a well-described epigenetic regulator in immune cells, is also expressed at high levels in RA-FLSs upon stimulation with proinflammatory stimuli such as IL-1β.^80^ It was proposed that miR-146a orchestrates the inflammatory response, suppressing the proliferation, changing the metabolic state and restricting the osteoclastogenic potential of RA-FLSs. These actions are elicited by targeting TNF receptor associated factor 6 (TRAF6), an important component of the NF-κB pathway, and disrupting the receptor activator of NF-κB ligand (RANKL)/osteoprotegerin (OPG) ratio. MiR-146a deficiency has been demonstrated to be responsible for aggravated inflammatory joint damage in a model of TNF-driven arthritis.^84^ Likewise, miR-146a potently inhibits the secretion of inflammatory factors as well as connective tissue growth factor (CTGF) by RA-FLSs. Downregulated CTGF expression limits the proliferation of RA-FLSs and angiogenesis, thereby, to some extent, limiting pannus formation and attacking cartilage.^85^ The levels of miR-143 and miR-145 are also augmented in RA-FLSs compared to OA-FLSs.^86^ Overexpression of miR-143 enhances TNFα-induced pro-inflammatory signals by downregulating insulin-like growth factor binding protein 5 (IGFBP5). MiR-145 regulates semaphorin 3 A (SEMA3A) to render RA-FLSs susceptible to vascular endothelial growth factor 165 (VEGF165) stimulation, contributing to cell migration and invasion.^86^ In addition, it has been noted that miR-145-5p exacerbates RA progression by promoting nuclear translocation of p65 to activate NF-κB, which increases the levels of MMP-9.^87^

Elevated expression of miR-21, miR-26a-5p, miR-203, miR-221, miR-222, miR-323-3p and miR-346 in RA-FLSs has also been reported. Upregulated miR-26a-5p was found to promote the proliferation, invasion, and apoptosis tolerance of RA-FLSs through regulation of the PTEN/PI3K/AKT pathway.^88^ MiR-21 facilitates NF-κB nuclear translocation to activate the NF-κB pathway, resulting in enhanced proliferation of RA-FLSs.^89^ The robust expression of miR-203 in RA-FLSs, which relies on the regulation of hypomethylation, seems to increase the release of MMP-1 and IL-6 by controlling NF-κB pathway activity.^90^ Using miR-seq-based profiling, Pandis et al. were the first to identify miR-221/222 and miR-323-3p as overexpressed miRNAs in FLSs from human TNF transgenic mice and patients with RA. The predicted target genes of miR-221/222 and miR-323-3p exhibited considerable enrichment in a number of RA-related pathways. Importantly, miR-323-3p was suggested to be strongly implicated in RA pathogenesis due to its positive modulation of the WNT/cadherin signaling pathway.^91^ Furthermore, induced expression of miR-346 was observed in LPS-activated RA-FLSs. The expression of a nonreceptor tyrosine kinase related to the stabilization of multiple inflammatory cytokines, named Bruton’s tyrosine kinase, is indirectly repressed by miR-346 upregulation, thus mediating the inhibition of IL-18 release in RA-FLSs.^92^

In contrast, several pivotal miRNAs were found to be significantly reduced in RA-FLSs. Najm et al. demonstrated the anti-inflammatory and anti-erosion role of miR-17-5p in both RA-FLSs and CIA mice, which was associated with reduced IL-6 secretion, decreased immune cell infiltration and osteoclastogenesis inhibition through the JAK1-STAT3 pathway.^93^ Lin et al. concluded that low miR-22 levels in RA-FLSs enhance the expression of cysteine-rich protein 61 (Cyr61), which is a component of the ECM, contributing to synovial hyperplasia and pannus formation. Intriguingly, whereas wild-type (WT) p53 activates miR-22 transcription by binding to its promoter region, the mutant p53 frequently observed in RA synovial tissues lacks the ability to upregulate the expression of miR-22, leading to Cyr61 deficiency. This further supports the concept that somatic p53 mutations are one of the etiological factors of RA.^94^ Nakamachi et al. showed that forced expression of miR-124a in RA-FLSs led to suppression of cell proliferation and G1 phase arrest because miR-124a bound to the 3′-UTR of cyclin-dependent kinase 2 (CDK-2) and monocyte chemoattractant protein 1 (MCP-1) mRNA.^95,96^ Of note, the expression of miR-124a is dictated by the methylation status of its encoding gene. Consistent with what has been reported in tumor cells, the miR-124a gene promoter was found to be hypermethylated in RA synovial tissue samples from patients with RA but nonmethylated in samples from healthy subjects.^97^ As such, it is not surprising that 5-aza-2ʹ-deoxycytidine (5-AzadC) treatment in vitro can inhibit the proliferation of RA-FLSs and the synthesis of TNF-α via demethylation of the miR-124a gene.^98^ More recently, Wang et al. revealed that overexpression of forkhead box protein C1 (FoxC1) in RA-FLSs and CIA rats, which is negatively correlated with miR-141-3p expression, might promote the proliferation, migration, invasion, and inflammatory cytokine secretion of RA-FLSs. The authors also revealed that FoxC1 interacts directly with β-catenin to activate the canonical Wnt signaling pathway.^99^ In addition, differential expression of both miR-9 and miR-106b in RA-FLSs leads to shifts not only in the invasive properties of RA-FLSs but also in RANKL levels, which affects osteoclastogenesis.^100,101^

Decreased expression in RA‑FLSs was also reported for miR-19, miR-20a, miR-27a, miR-29a, miR-152, miR192, miR‑199a-3p, miR‑375, miR-34a, miR-15a/16, and miR-3926. MiR-19a/b can act as a negative regulator of the inflammatory response in RA-FLSs, which is supported by evidence showing that miR-19a/b diminishes IL-6 and MMP-3 production via direct control of TLR2 expression.^102^ MiR-27a inhibition in RA-FLSs significantly promotes cell migration and invasion by targeting the 3′ UTR of follistatin-like protein 1 (FSTL1) and further interfering with TLR4/NF-κB signaling.^103^ STAT3 and caveolin 1 (CAV1) have been identified as direct targets of miR-29a and miR-192 in RA-FLSs, respectively. MiR-29a-mediated STAT3 suppression and miR-192-mediated CAV1 inhibition can markedly restrict the expansion of RA-FLSs by reducing proliferation and triggering apoptosis. MiR-29a also has an anti-inflammatory role in RA-FLSs since it improves inflammatory cytokine levels.^104,105^ Moreover, decreased miR-199a-3p expression in RA-FLSs contributes to increased cell numbers. The growth-suppressive effects of miR-199a-3p were elicited by direct endogenous retinoblastoma 1 (RB1) inhibition, which resulted in an increase in caspase-3 activity and the Bax-Bcl-2 ratio.^106^ MiR-375 in RA-FLSs showed protective effects against synovial pathological changes that occurred as a result of decreased MMP-3 and fibronectin levels, as well as canonical Wnt signaling inactivation.^107^ Song et al. demonstrated substantially lower expression of miR-34a-5p in synovial tissues from patients with RA than in those from patients with OA, accompanied by robust proliferation of RA-FLSs and high TNF-α and IL-6 expression.^108^ Likewise, Wu et al. highlighted the therapeutic role of miR-34a-containing bone marrow mesenchymal stem cell (BMSC)-derived miR-34a in RA-FLSs. The authors’ investigation indicated that miR-34a ameliorates abnormal RA-FLS proliferation and inflammation by targeting cyclin I and further activating ATM/ATR/p53 signaling.^109^ Moreover, miR-20a, miR-15a/16 and miR-3926 negatively regulate the aggressive behavior of RA-FLSs and synovial inflammatory infiltration. The target genes of these miRNAs in RA-FLSs have been confirmed in various studies.^110–112^

### MiRNA-mediated dysfunction in immune cells

Previous studies have proven that miRNAs function as ‘fine-tuners’ of the expression of proteins involved in the immune response. Multiple miRNAs with immunoregulatory effects ensure the development and normal function of the immune system as well as the maintenance of immune homeostasis by establishing a balance between activation and inhibition.^113^

#### Disturbed monocyte-macrophage lineage function

Research has found that the expression profiles of miRNAs are highly variable in distinct macrophage polarization states.^114^ As an advantage, the temporal and spatial specificity of miRNA expression patterns provides flexible adjustment capacity to meet the high plasticity of macrophages during the response to environmental stimuli. Over the past decade, the functional roles of miRNAs in many aspects of monocyte/macrophage biology, particularly in macrophage polarization, have been intensively investigated. Dysregulation of miRNA levels thus appears to contribute to RA pathogenesis by perturbing the normal function of the monocyte-macrophage lineage (Table 1).

**Table 1:** MiRNAs in the monocyte-macrophage lineage in RA

MiRNA	Expression status in RA samples	Target(s)	Function(s)	Ref.
MiR-155	Increased	SHIP1, SOCS1	↑Inflammation ↑ Monocyte recruitment and retention ↓ Apoptosis	^115,120,121,124,125^
MiR-146a	Increased	Notch1, PPARγ, TRAF6, IRAK1	↓ Inflammation ↓ M1 polarization ↑ Myeloid cell differentiation/maturation	^116,117,119,123,127^
MiR-223	Increased	STAT3, TRAF6, Pknox1, NLRP3, AHRT	↓ M1 polarization ↑ M2 polarization ↑ AHR-mediated repression of pro-inflammatory cytokines	^130–134^
MiR-221-3p	Increased	JAK3	↑M1 polarization ↑ Inflammation	^135^
MiR-125a-5p^a^	Increased	KLF13, TRAF6, NEAT1	↓M1 polarization ↑ M2 polarization ↓ Inflammation	^136,137,294^
MiR-144-5p^a^	ND	TLR2	↓Inflammation ↑ Cell viability	^138^
MiR-17^a^ MiR-20a^a^ MiR-106a^a^	Decreased	SIRPα	↑ Macrophage activation, infiltration, phagocytosis ↑ Inflammation	^112,139,295,296^
MiR-33^a^	Increased	NLRP3	↑ROS accumulation ↑ Inflammation	^145^
MiR-30a	Decreased	NLRP3	↓Inflammation	^146^
MiR-let-7a^a^	Decreased	K-Ras, HMGA2	↓ Macrophage activation ↓ Inflammation	^147^ ^148^
MiR-let-7b	Increased	TLR-7	↑M1 polarization	^149^

*miRNA* microRNA, *RA* rheumatoid arthritis, *ND* not determined

^a^As described in in vitro cellular models; no data in animal models mimicking RA conditions were available

Two extensively studied miRNAs, miR-155 and miR-146a, are strongly expressed in RA macrophages and exert proinflammatory and anti-inflammatory effects, respectively. Defective differentiation of circulating monocytes from patients with RA into M2 macrophages is associated with increased miR-155 expression.^115^ Conversely, miR-146a is differentially expressed in M1 macrophages and M2 macrophages and endows macrophages with the potential to preferentially differentiate toward an M2 phenotype by regulating the expression of Notch1 and PPARγ.^116,117^ Exposure to a variety of inflammatory stimuli or direct recognition of microbial components such as lipopolysaccharide (LPS) by TLRs regulates the expression of both miR-155 and miR-146a in macrophages in an NF-κB-dependent and temporally asymmetric manner.^118,119^ Mann et al. unveiled a combined positive and negative regulatory network controlling NF-κB signaling activity in which miR-155 expression is induced prior to miR-146a expression and plays a dominant role in the promotion of the inflammatory response.^120^ Mechanistically, miR-155 ensures robust NF-κB signaling activity via Src homology 2-containing inositol phosphatase-1 (SHIP1) and suppressor of cytokine signaling 1 (SOCS1) repression.^120,121^ TRAF6 and IL-1 receptor-associated kinase 1 (IRAK1), the encoded proteins of which are located downstream of TLR signaling, have been identified as target genes for posttranslational repression by miR-146a. MiR-146a, therefore, constitutes an essential part of a negative feedback loop in macrophages that attenuates the activation of NF-κB signaling and reduces the production of inflammatory factors.^119^ Nevertheless, since miR-146a expression in macrophages reaches saturation at sub-inflammatory levels,^122^ it appears that the abnormal miR-146a expression in RA is not sufficient to counteract the positive effect of miR-155 on NF-κB activity, thus contributing to the chronicity of inflammation. This idea was further verified in experimental CIA mice established by Nakasa et al., in which transfection of miR-146a, despite inducing partial relief, failed to eliminate synovial inflammation.^123^

MiR-155 also participates in the development of RA by promoting monocyte recruitment and retention at inflammation sites. Upregulation of miR-155 in RA monocytes not only diminishes their susceptibility to apoptosis^124^ but also augments proinflammatory chemokine production and decreases chemokine receptor expression.^125^ Mice lacking miR-155 are resistant to the development of articular inflammation.^126^ Furthermore, Ghani et al. revealed that the expression of miR-146a, regulated by PU.1, is indispensable for driving the myeloid cell differentiation/maturation process.^127^ Given the characteristic elevation of miR-146a expression in THP-1 cells in a tolerized state, miR-146a is also thought to play pivotal roles in endotoxin tolerance and endotoxin-induced cross-tolerance, suggesting that it has potential functions in preventing overreactive inflammation.^128^ When compared with WT controls, serum transfer arthritis (STA) K/BxN mice with miR-146a deletion show a statistically significant increase in the arthritis index.^129^

Increased expression levels of miR-223 have been shown in various compartments, such as plasma, serum, synovial tissues, and synovial fluid, in patients with RA. Many research teams have recognized the essential role of miR-223 in regulating macrophage polarization. For instance, miR-223 downregulation promotes the release of IL-6 and IL-1β by activating STAT3^130^ and improves the M1/M2 macrophage ratio by targeting TRAF6 and subsequently influencing the NF-κB signaling pathway.^131^ Zhuang et al. identified Pknox1 as another target gene of miR-223. Augmented miR-223 expression mediates the inhibition of Pknox1 and further fosters M2 polarization.^132^ Moreover, nucleotide-binding domain-(NOD)-like receptor family, pyrin domain containing 3 (NLRP3) inflammasome activity, which is reported to be associated with the initiation, progression and perpetuation of RA, is negatively controlled by miR-223 in macrophages, leading to decreased IL-1β production.^133^ However, an opposing perspective was raised by Ogando and coworkers. The authors validated that miR-223 is upregulated in RA-derived macrophages compared to OA-derived macrophages and reported that high miR-223 levels prevent aryl hydrocarbon receptor (AHR) activation in myeloid cells by reducing AHR nuclear translocator protein levels. Thus, miR-223 in macrophages appears to exhibit proinflammatory effects due to the repression of LPS-induced proinflammatory cytokine expression after AHR agonist treatment.^134^ Despite the controversy, the promising potential of miR-223 in modulating macrophage function can be seen in the growing list of its confirmed target genes. More data are needed to reveal the dual roles of miR-223 in macrophages, especially in RA disease conditions.

Forced high expression of miR-221-3p after TLR4 activation interfered with JAK3/STAT3 signaling in M2 macrophages, driving M2 macrophages to exhibit an M1 phenotype. This dysregulated balance of M1/M2 macrophage populations contributed to the proinflammatory cytokine/chemokine secretion profile in RA.^135^ In contrast, higher expression of miR-125a-5p was detected in M2 macrophages than in M1 macrophages. Elevated levels of miR-125a-5p after TLR2 or TLR4 activation promote alternative activation of macrophages, pointing toward an anti-inflammatory role of this miRNA.^136^ Administration of miR-125a-5p mimics in LPS-induced macrophages leads to diminished TRAF6 expression and TAK1 protein phosphorylation, which is correlated with an elevated proportion of M2 phenotype macrophages.^137^ In addition, Zhou et al. indicated that miR-144-5p overexpression suppresses the secretion of TNF-α, IL-6 and IL-8 in LPS-treated THP-1 macrophages concomitant with decreased cell activity by inhibiting TLR2 expression and impairing the NF-κB signaling pathway.^138^ MiR-17, miR-20a, and miR-106a potentiate phagocytosis and the inflammatory response of macrophages by posttranscriptionally reducing signal-regulatory protein α (SIRPα) synthesis.^139^

Clinical evidence has demonstrated that the NLRP3 inflammasome plays a crucial role in multifarious diseases, including RA.^140,141^ Further studies revealed that the NLRP3 expression level was associated with clinical arthritis severity and the extent of radiological destruction.^142,143^ In particular, activation of the NLRP3 inflammasome occurs mainly in the monocyte-macrophage lineage.^144^ The miRNAs that participate in RA pathogenesis through NLRP3 inflammasome activation have been broadly researched. Xie et al. stated that both miR‑33 levels and NLRP3 inflammasome activity were increased in clinical samples from patients with RA. Mechanistically, miR‑33 overexpression resulted in the accumulation of cellular ROS and enhanced NLRP3 levels, caspase‑1 activity and IL‑1β production in primary macrophages, thereby eliciting NLRP3 inflammasome activation.^145^ Yang et al. were the first to demonstrate that knockout of NLRP3 or intra-articular injection of miR-30a achieved significant therapeutic effects in TNF^TG^ mice by remarkably ameliorating inflammatory joint destruction. Subsequently, functional studies further proved that miR-30a directly interacts with the 3’ UTR of NLRP3 and mediates NLRP3 inhibition.^146^

Decreased miR-let-7a expression was observed in monocytes from ACPA-positive RA patients. MiR-let-7a downregulation augments the inflammatory response by increasing ACPA-mediated ERK1/2 and JNK phosphorylation and inducing IL-1β release via Ras proteins.^147^ In addition, Zhu et al. reported that miR-let-7a can inhibit ACPA-induced macrophage activation by directly targeting HMGA2.^148^ In contrast, high expression of miR-let-7b provokes arthritic joint inflammation. MiR-let-7b has been recognized as a novel endogenous ligand for joint TLR-7. Interruption of miR-let-7b ligation to TLR-7 obviously impairs the M1 polarization of synovial macrophages in RA.^149^

#### Disturbed CD4^+^ T-cell function

**Fig. 5:**
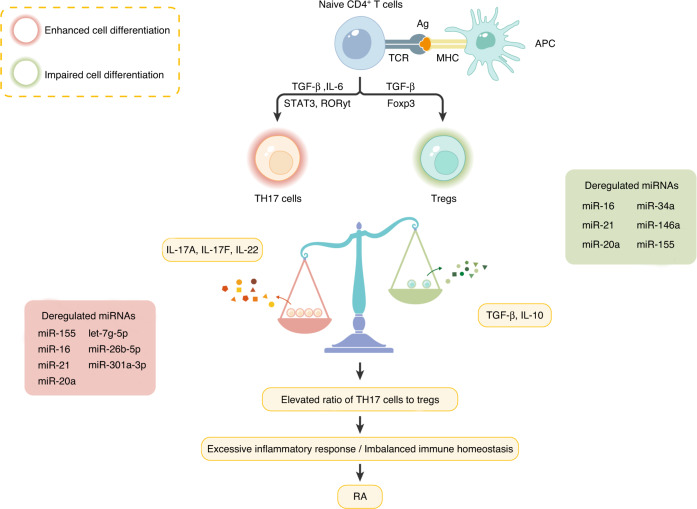
Deregulated miRNAs in RA that affect the Th17/Treg ratio. One of the central events in RA pathology is the imbalance between Th17 cells and Tregs. In the context of RA, activated APCs capture and process autoantigens for subsequent presentation to naïve CD4^+^ T cells, which provokes CD4^+^ T-cell activation and differentiation. The differentiation and expansion of Th17 cells are enhanced, leading to overwhelming inflammation via excessive inflammatory cytokine (for example, IL-17) release. In parallel, the Treg proportion is decreased, and the suppressive properties of Tregs are impaired, which is correlated with disrupted immune homeostasis. A consequent increase in the Th17/Treg ratio accelerates RA progression. The differential expression of several miRNAs changes the frequency and function of Th17 cells (red box) and/or Tregs (green box), thereby promoting the pathogenic role of these cells in RA. miRNA microRNA, RA rheumatoid arthritis, Th17 cell T helper 17 cell, Treg regulatory T cell, APC antigen-presenting cell

Studies have shown pivotal roles of miRNAs in regulating the cellular processes of CD4^+^ T cells (Fig. 5).

The differentiation and activation of CD4^+^ T-cell subpopulations and their effector functions may be jointly controlled by the different miRNAs they express. Classically, RA has been thought of as a disease that is mediated by interferon-γ (IFN-γ)-producing Th1 cells. A series of early studies proposed some miRNAs as crucial regulators of the Th1 immune response. For instance, activated CD4^+^ T cells are observed to induce miR-155 expression, which facilitates Th1 differentiation by modifying the IFN-γRα chain.^150^ The miR-17-92 cluster is also involved in modulating the Th1 response, which is directed almost entirely by specific individuals within this cluster. Specifically, miR-17 and miR-19b act in synergy to promote the functions of Th1 cells, with three functional targets: TGFβRII, CREB1 and Pten.^151^ Conversely, Th1 differentiation is inhibited while abnormal IFN-γ production is corrected by miR-29.^152^

However, the Th1 cell subset is not responsible for all the mechanisms underlying RA based on previously published studies. In recent years, the role of proinflammatory Th17 cells in RA has drawn increasing attention. Patients with RA often have enhanced Th17 cell differentiation, which is at the expense of Treg differentiation, and these integrated changes favor systemic inflammation in RA.^5,20,153^ Jarid2 is a DNA-binding protein that recruits Polycomb repressive complex 2 (PRC2) to bind to chromatin and hence mediates silencing of cytokine genes in Th17 cells. During Th17 differentiation, miR-155 is highly expressed and contributes to the activation of Th17-related cytokine gene expression by decreasing Jarid2 expression.^154^ Correspondingly, mice deficient in miR-155 demonstrated decreased sensitivity to CIA and profoundly impaired Th17 polarization.^126^ There is a rich supply of data pointing to miR-155’s pathogenic role in both the innate and adaptive immune systems. Intriguingly, miR-155 is also essential for the maintenance of Treg homeostasis. Despite contributing to the suppressive function of Tregs, high expression of miR-155 also ensures increased Treg generation in cases of lymphodepletion by targeting SOCS1, a JAK-STAT signaling inhibitor.^155,156^ Given the limited number and controversial results of published studies, much uncertainty exists about the levels of miR-155 in Tregs its regulatory effect on the proportion of Tregs in RA. Elevated miR-155 expression in RA Tregs was observed by Kmiolek and coworkers,^157^ whereas Zhou and coworkers showed a decrease in miR-155 expression in RA Tregs.^158^ Further investigations focusing on specific cell subsets rather than entire populations are warranted to provide a comprehensive understanding of the role of miR-155 in RA immunity.

Another miRNA that is tightly associated with the normal status of Tregs is miR-146a. Unlike the case for miR-155, high miR-146a levels are required for Treg suppression of autoimmunity. MiR-146a in Tregs functions by directly restraining STAT1 activation rather than by impacting NF-κB signaling in myeloid cells.^159^ Robust expression of miR-146a, however, is restricted to Tregs of RA patients. MiR-146a deletion in Tregs of patients with active RA leads to a proinflammatory phenotype of Tregs, accompanied by intensive inflammatory cytokine secretion. Strikingly, the extent of this restriction was found to be positively correlated with disease activity, implying that miR-146a might maintain high expression levels in Tregs of healthy individuals or even in Tregs of patients with low disease activity.^158^

The ratio of Th17 cells to Tregs is also affected by ectopic expression of other miRNAs. The expression of miR-301a-3p in peripheral blood monocytes (PBMCs) from RA patients was higher than that in PBMCs from healthy controls. Upregulation of miR-301a-3p increased the levels of STAT3 by targeting protein inhibitor of activated STAT3 (PIAS3), resulting in a larger Th17 cell population.^160^ As an essential transcription factor involved in Th17 cell differentiation, STAT3 directly induces the production of IL-17, a representative cytokine of Th17 cells.^161^ Another miRNA, let-7g-5p, was recognized to be inversely correlated with the proportion of Th17 cells, and its expression is repressed in RA patients and CIA mice.^162^ A recent study proposed that let-7g-5p overexpression, which disrupts STAT3 expression, alleviates CIA symptoms and predominantly hinders the transdifferentiation of Tregs into Th17-like cells.^163^ Increased miR-26b-5p expression was also demonstrated to have a positive effect on CIA. The difference is that this was accomplished mainly by perturbing the conventional differentiation of Th17 cells.^164^ In the context of RA, the NF-κB signaling pathway activated by IL-6 and TNF-α from the inflammatory milieu triggers the sustained expression of miR-34a, reducing the levels of the transcription factor FoxP3, which is specifically expressed in Tregs and critically governs their development and function.^165^ Furthermore, some miRNAs display simultaneous effects on the differentiation of Th17 cells and Tregs. The disrupted Th17/Treg balance caused by aberrant miR-20a expression was suggested to be closely related to the activation of the NLRP3 inflammasome.^166^ MiR-21 deficiency in RA patients promotes the pathogenicity of Th17 cells via potent STAT3 expression and mediates Treg dysfunction via reduced FoxP3 levels.^153^ Jin et al. identified Maresin 1 (MaR1) as a mediator upstream of miR-21 that upregulates its expression. Not surprisingly, by modifying MaR1 in the CIA mouse model, the authors demonstrated that miR-21 decelerated RA progression and restored the appropriate Th17/Treg ratio.^167^ MiR-16 was found to maintain high levels in Th17 cells, while miR-16 levels were low in Tregs. This differential expression controls the levels of the transcription factors RORγt and FoxP3 at the transcriptional and translational levels, contributing to an elevated Th17/Treg ratio.^168^

Expansion of Tfh cells in RA orchestrates spontaneous germinal center (GC) formation and excessive autoantibody synthesis.^169^ There are limitations to existing data regarding the role of individual miRNAs in Tfh cell subsets. Among the relevant studies, the two with the strongest conclusions regarding the importance of miRNAs in the functional regulation of Tfh cells were performed on miR-146a and miR-155. Pratama et al. highlighted that upregulation of miR-146a in Tfh cells decreases their numbers and restricts Tfh cell responses, in line with the widely discussed role of miR-146a as an intracellular molecular brake in the orchestration of universal biological processes.^170^ In contrast, miR-155 was found to counterregulate the proliferation and differentiation of Tfh cells.^171^ Considering that knockdown of miR-155 completely reverses the autoimmune pathology in mice with miR-146a deletion, a miR-155-dependent mechanism is suggested to underly the uncontrolled accumulation of Tfh cells induced by loss of miR-146a.^172^ In view of the reported fundamental roles of miR-155 and miR-146a in Tfh cell development, whether these two important miRNAs exert their putative effects on Tfh cells in induced arthritis models or RA patients through predictable or unpredictable molecular mechanisms needs to be determined.

#### Disturbed B-cell function

Although many miRNAs have been implicated in the determination of B-cell fates, few have been validated to have clear effects within a specific disease model of RA. MiR-155 is the only miRNA that has been previously shown to be prevalently expressed in peripheral blood and synovial fluid CD19^+^ B cells and synovial tissue of RA patients. Overexpression of this miRNA induced by PU.1 inhibition was shown to positively regulate B-cell activation in RA, implying subsequent persistent production of autoantibodies.^173^ More recently, an in-depth analysis of miRNA expression profiles in blood CD19^+^ B cells revealed differences in the expression of 27 miRNAs between RA patients in remission treated with methotrexate (MTX) and healthy controls, and these miRNAs were predicted to target genes known to be crucial for B-cell development.^174^

#### Disturbed dendritic cell (DC) function

An miR-363/integrin αv/TGF-β axis associated with the promotion of Th17 cell polarization was identified in DCs from patients with RA.^175^ Analogously, it was proposed that forced expression of miR-34a facilitates DC activation in RA by targeting AXL. Mouse models with miR-34a deficiency are resistant to CIA and may not support the development of Th17 cells due to diminished DC-T-cell interactions in vivo.^176^ In addition to the suppressive effect of miR-34a on RA Tregs we discussed before, miR-34a also seems to serve as a dual regulator causing significant Th17/Treg ratio imbalance, which accelerates the progression of RA.

#### Disturbed neutrophil function

Rosa et al. noted impaired miRNA biogenesis in neutrophils from RA patients, as reflected by a global decline in miRNA levels that led to the pathogenic activation of neutrophils.^177^ Typically, Murata et al. reported that decreased expression of miR-451 in RA neutrophils contributes to their chemotaxis toward inflammation sites, which is induced by 14-3-3ζ- and Rab5a-mediated p38 MAPK phosphorylation. The authors suggested CPNE3 and Rab5a as two novel target genes directly silenced by miR-451.^178^ Nevertheless, as p38 MAPK signaling pathway activity showed no correlation with CPEN3, the role of CPNE3 and miR-451 in RA neutrophils requires further verification.

### MiRNA-mediated dysfunction in bone-related cells

#### Disturbed osteoclast function

**Fig. 6:**
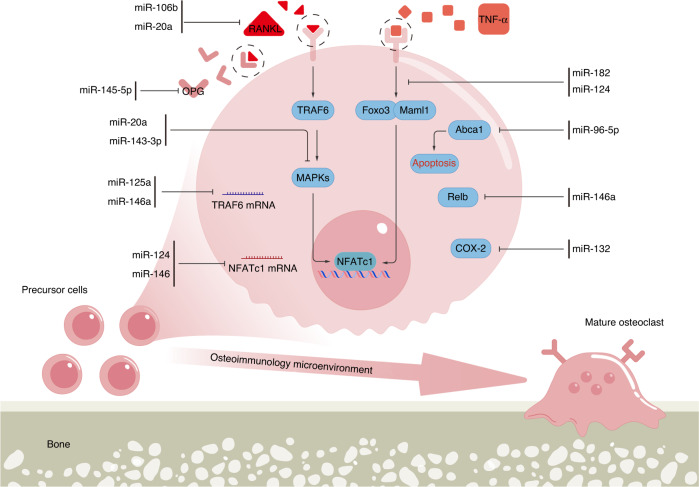
Deregulated miRNAs in RA affect osteoclastogenesis. Gross disturbances in skeletal structure and function in synovial joints of patients with RA are mostly the result of an imbalance in osteoclast-mediated bone remodeling. The discovery of RANKL signaling was indispensable for an in-depth understanding of the mechanisms underlying osteoclastogenesis and bone resorption. Dysregulated miRNA expression in RA, driven by the osteoimmune microenvironment, is capable of controlling the differentiation and maturation of osteoclasts through RANKL-dependent (left) and RANKL-independent (right) pathways, resulting in a progressive reduction in periarticular bone mass and subsequent bone erosion. miRNA microRNA, RA rheumatoid arthritis, RANKL receptor activator of nuclear factor-kappa B ligand

MiRNAs have been implicated in the biological regulation of osteoclast function.^179^ Pre-miRNAs are cleaved to duplex mature miRNAs by the endonuclease RNase III Dicer, and osteoclast-specific Dicer gene deficiency was proven to suppress osteoclastic bone resorption.^180^ Accordingly, dysregulated miRNA expression modulates osteoclast development and function, leading to the inhibition or promotion of bone destruction in RA (Fig. 6).

##### RANKL-dependent osteoclastogenesis signaling pathways

RANKL-dependent osteoclastogenesis signaling pathways are the main regulatory pathways related to overactivation of osteoclasts in bone disorders.^37^ Numerous miRNAs have been shown to be differentially expressed during osteoclast differentiation and exert crucial roles in RANKL-dependent osteoclast activation in RA.

First, the binding of RANKL to RANK largely regulates initial osteoclast precursor differentiation and is considered the trigger in osteoclast activation. The density of RANK on the cell surface and the local levels of RANKL affect the strength of RANKL/RANK signaling and downstream signal transduction.^36^ MiR-106b and miRNA-20a were found to inhibit RANKL expression by directly binding to the 3′UTR of RANKL mRNA, blocking osteoclast differentiation.^181,182^ One of our previous studies revealed that inhibition of miR-106b substantially repressed mature osteoclast formation in vitro and alleviated the pathologic process of arthritis in CIA mice, accompanied by a decreased RANKL/OPG ratio.^183^ Kong et al. also demonstrated that miR-20a agomir injection impeded osteoclast proliferation and differentiation and thus improved bone erosion in a CIA mouse model.^182^

The binding of RANKL to RANK induces the recruitment of the adaptor protein TRAF6, and this process is controlled by miR-125a and miR-146a.^119,184^ MiR-125a expression is dramatically downregulated during osteoclast differentiation. Overexpression of miR-125a restricted osteoclastogenesis because miR-125a targets TRAF6, whereas the transcription of miR-125a was negatively regulated by the binding of NFATc1 to the promoter of miR-125a, suggesting the existence of a TRAF6/NFATc1/miR-125a regulatory feedback loop.^184^ In a CIA rat model, miR-125a treatment effectively attenuated arthritis severity.^185^ Moreover, miR-146 was reported to blunt TLR and cytokine signaling by base-pairing with sequences in the 3′ UTRs of TRAF6.^119^

The RANK/TRAF6 signaling cascade activates the MAPK pathway, potentially leading to additional miRNA targets. Forced expression of miR-20a inhibited the proliferation and differentiation potential of RA osteoclasts via the TLR4/p38 pathway.^182^ Abnormally low expression of miR-143-3p was noted in both clinical and experimental samples from RA patients and arthritis animals. Importantly, treatment with MAKP inhibitors elevated miR-143-3p levels in osteoclasts from adjuvant-induced arthritis (AIA) rats. Upregulated miR-143-3p expression was found to mediate osteoclastogenesis suppression by diminishing the expression of the early-stage protein ERK1/2 and the late-stage protein JNK in MAPK signaling.^186^ In the study, the authors first investigated the differential expression of miR-143-3p in vivo and further verified the regulatory mechanism of osteoclast differentiation in vitro. The limitation is that the detailed therapeutic effects of miR-143-3p administration on bone erosion in arthritic animal models were not investigated.

Another pivotal downstream signaling pathway activated by the RANK/TRAF6 signaling cascade is the NF-κB pathway. The activation of NF-кB signaling is involved in the regulation of a wide range of pathological processes during RA, such as synovitis and immune dysfunction. de la Rica et al. demonstrated that NF-κB directs monocyte-to-osteoclast conversion through an miRNA-dependent mechanism. NF-κB p65 can bind the promoters of two miRNA clusters: miR-212/132 and miR-99b/let-7e/125a. The NF-κB-directed activation of these miRNAs downregulates the expression of certain monocyte-specific and immunomodulatory genes, mediating proper osteoclast differentiation.^187^ Of note, contradictory outcomes emerged when validating the efficacy of these two NF-κB-associated miRNA clusters in animal models of RA. Articular knockdown of miR-132 in AIA murine models ameliorated bone erosion in the joints,^188^ while miR-125a overexpression retarded arthritis progression in CIA rats.^185^ These contradictory results might be explained by the complicated molecular mechanisms of miRNAs in different cell types and cell developmental stages. However, whether miR-132 and miR-125a are associated with NF-κB pathway activity in osteoclasts in the setting of RA is still unclear. Although growing data have shown a significant correlation between NF-кB signaling and deregulated miRNA levels in RA, strikingly, there is a lack of knowledge on whether specific miRNAs affect osteoclast differentiation during RA via NF-κB modulation. Follow-up investigations should take into account the cell specificity and condition specificity of the cellular signaling pathway.

NFATc1 has recently been described as a direct target of miRNAs. Consistent with previous findings regarding the NFATc1-dependent suppressive effects of miR-124 on osteoclastogenesis,^189^ forced expression of miR-124 in the ankle joints of AIA rats attenuated bone erosion, and direct binding of miR-124 to the 3’UTR of NFATc1 was identified.^190^ Interestingly, despite the inability of miR-124 to target RANKL mRNA, the mRNA and protein expression of RANKL was reduced in the ankle tissues of rats treated with pre-miR-124.^190^ MiR-146a also exerts inhibitory effects on osteoclast differentiation of human PBMCs in a dose-dependent manner by reducing NFATc1, c-Jun, and TRAP expression. Intravenous administration of double-stranded miR-146a resulted in the suppression of cartilage and bone destruction in CIA mice.^123,191^ Further studies should be performed to validate the direct binding of miR-146a to the 3′ UTR of NFATc1 mRNA.

OPG binds with strong affinity to RANKL, which limits bone resorption by competitively preventing RANKL from binding to its receptor RANK. MiR-145-5p not only strongly participates in RA-FLS pathology as mentioned above but also exerts destructive functions in osteoclasts. Augmented miR-145-5p expression in osteoclasts leads to a significant increase in RANK and RANKL expression and a decrease in OPG levels in a time-dependent manner. OPG has been identified as a target of miR-145-5p in osteoclasts.^192^ Intravenous injection of an miR-145-5p agomir resulted in more severe joint damage in a CIA model.^192^

##### RANKL-independent osteoclastogenesis signaling pathways

Recent discoveries have also revealed the essential role of alternative pathways in RANKL-independent osteoclastogenesis. TNF, as an important cytokine in the pathophysiology of RA, can also elicit osteoclast differentiation.^193^ Ohnuma et al. explored the potential role of miR-124 in the RANKL-independent osteoclastogenesis pathway. The researchers suggested that miR-124 represses osteoclast differentiation in response to stimulation with TNF-α, IL-6, and M-CSF. The expression levels of osteoclast-specific genes and NFATc1 protein were reduced in miR-124 mimic-transfected osteoclasts.^194^ Miller et al. demonstrated that miR-182 is also a negative regulator of TNF-α-mediated osteoclastogenesis via regulation of Foxo3 and Maml1, which may reveal an effective therapeutic approach in RA.^195^ More in vivo experimental evidence is still urgently needed for translation of relevant miRNA-related strategies into the clinic. With the application of anti-TNF drugs in clinical practice, future studies may focus on whether miRNA-based therapies can act synergistically with anti-TNF therapy in RA.

The mitochondrial apoptosis pathway can regulate the proliferation and activity of osteoclasts.^196^ Reduced miR-96-5p expression in osteoclasts mediates the regulation of ATP binding cassette subfamily A member 1 (Abca1), contributing to an increased level of mitochondria-dependent apoptosis in vitro and attenuated arthritic bone loss in vivo.^197^

During osteoclast differentiation, miR-146a hinders osteoclast progenitor differentiation through a marked decrease in Relb transcript levels. Targeted delivery of miR-146a into Ly6C^high^ monocytes was shown to effectively repress monocyte-to-osteoclast conversion and prevent joint destruction in CIA mice.^129^

#### Disturbed osteoblast function

MiRNAs have emerged as major regulators of osteogenesis.^179^ Maeda et al. identified 22 deregulated miRNAs in the inflamed synovium of serum-transfer arthritis K/BxN mice related to bone formation, and the putative candidate targets are components of the Wnt and bone morphogenetic protein (BMP) pathways.^198^ MiR-218 establishes a positive feedback loop in osteoblasts to promote osteogenic differentiation.^199^ Specifically, miR-218 is abundantly expressed in response to canonical Wnt signaling. By suppressing multiple Wnt antagonists, such as DKK proteins, overexpression of miR-218 in turn reinforces Wnt signaling. Of interest, it appears that activation of Wnt signaling by miR-218 also increases the osteogenic differentiation of RA-FLSs, which contributes to the repair of bone erosion in RA.^200^ A limitation of current studies on miRNAs regulating osteoblast-mediated bone formation in RA is that most of them have only validated diverse miRNA–mRNA interactions using in vitro experiments. Sufficient evidence to prove the therapeutic efficacy of these miRNAs in RA patients and/or animal models is still lacking, making clinical translation challenging.

It has been proposed that Akt signaling cascade activation leads to miR-93, miR-129-3p, and miR-590-3p depletion in RA osteoblasts.^201–203^ Downregulated miR-93 expression mediates induction of oncostatin M, which is a pleiotropic cytokine of the IL-6 family that participates in RA pathogenesis.^201^ Loss of miR-129-3p in osteoblasts directly induces IL-17 protein synthesis at the posttranscriptional level and facilitates monocyte migration during RA.^202^ As precursors of macrophages, osteoclasts and DCs, monocytes tend to commit to a certain differentiation pathway based on the surrounding conditions upon migration into localized tissues. Massive monocyte infiltration in inflamed joints of RA is therefore sufficient to support the inflammatory reaction and bone destruction. Hyperplastic pannus nourished by neovascularization is a hallmark of RA, and prior research has revealed coupling of osteogenesis and angiogenesis.^204^ Due to the direct interaction of miR-590-3p with IL-18 mRNA, a low level of miR-590-3p in osteoblasts upregulates IL-18 expression and subsequently promotes the migration and tube formation of endothelial progenitor cells (EPCs).^203^ In addition, as Cyr61 is abundantly expressed in the synovial fluid of RA patients. This ECM protein is capable of inhibiting miR-126 and miR-518-5p expression in osteoblasts via the protein kinase C-alpha (PKC-α) and MAPK signaling cascades, respectively. In vitro functional research has clarified that miR-126 and miR-518-5p directly bind to the 3′ UTR of VEGF and the chemokine MCP-1 (also known as CCL2), respectively, leading to restriction of EPC angiogenesis and monocyte migration. Knockdown of Cyr61 in osteoblasts effectively prevents CIA progression and reduces disease severity.^205,206^ Similar to miR-126, in both in vitro and in vivo experiments, miR-16-5p was demonstrated to affect angiogenesis of EPCs by decreasing VEGF levels in osteoblasts. The expression of miR-16-5p in osteoblasts is regulated by c-Src-dependent FAK signaling.^207^

These findings characterize the tightly regulated expression patterns and pleiotropic effects of miRNAs. Aberrant miRNA expression profiles in RA osteoblasts control structural joint damage from multiple angles, impacting not only osteoblast differentiation but also cell chemotaxis and angiogenesis.

#### Disturbed chondrocyte function

An investigation launched by Chen et al. utilized NGS technologies and bioinformatics analyses to identify the expression profile of miRNAs in RA chondrocytes and to screen a series of candidate target genes.^208^ Overexpression of the long noncoding RNAs (lncRNAs) ZNF667-AS1, HOTAIR and MEG3 in LPS-induced chondrocytes stimulated chondrogenic proliferation and alleviated inflammation by regulating the production of inflammatory factors such as IL-6, IL-17 and IL-23, as well as the proliferation-related protein Ki67. Importantly, the protective effects of these three lncRNAs were effectively abrogated by miR-523-3p, miR-138, and miR-141, which participate in the activation of the JAK/STAT, NF-κB and AKT/mTOR signaling pathways, respectively.^209–211^ In a study in primary articular chondrocytes, miR-23a diminished IL-17-dependent proinflammatory mediator secretion, which was first proven to be mediated by direct interaction with IKKα, a kinase of the NF-κB pathway.^212^ Due to its induction of abnormal levels of type II collagen, MMP-13, COX-2, and IL-6, forced expression of miR-498 was suggested to alter the cellular signature via mTOR silencing in LPS-treated chondrocytes and thus aggravate ECM degradation.^213^ In addition, in vitro experiments conducted by Zhou et al. indicated that the arrest of chondrocyte apoptosis as a result of miR-27b-3p overexpression was related to the control of HIPK2 expression.^214^ Rats treated with interventions targeting miR-26a were shown to be resistant to arthritis in an experimental arthritis model established by Jiang et al. The authors also highlighted that high amounts of miR-26a regulate chondrogenic proliferation and apoptosis to increase cartilage destruction via CTGF repression.^215^

MiR-140, specifically expressed in cartilage, is fundamental to the maintenance of cartilage function and homeostasis.^216^ The regulatory role of miR-140 in chondrocytes and the underlying molecular mechanisms have been broadly investigated in OA.^217–219^ The aberrant synovial microenvironment governs cartilage damage in the context of RA, and an RA-FLS-based function of miR-140 has been identified.^220^ Consequently, it is reasonable to assume that miR-140 is also implicated in chondrocyte fate determination in RA. According to the study by Chen et al. mentioned above, deregulated miR-140-3p in RA chondrocytes might target FGF9, controlling the cell cycle to affect chondrogenic proliferation.^208^ This finding is in line with the results from several miRNA prediction databases, but further experimental verification is needed.

## Roles of exosomal MiRNAs in RA progression

Growing data have revealed the tight association of exosomal miRNAs with RA pathogenesis.^60^ In addition to being recognized as a novel class of endocrine factors for cellular self-regulation, miRNAs might also serve as paracrine messengers to establish intercellular communication in the context of RA. Recent studies exploring the cellular sources of exosomes in RA have focused on MSCs, synoviocytes, and leukocytes (Table 2).

**Table 2:** Exosomal miRNAs implicated in RA

Cellular origin	Encapsulated miRNAs	Recipient cell(s)	Target(s)	Function(s)	Ref.
FLSs	MiR-221-3p	Osteoblasts	DKK2	↓Osteogenesis	^198^
FLSs	MiR-486-5p	Osteoblasts	TOB1	↑Osteogenesis	^221^
FLSs	MiR-106b	Chondrocytes	PDK4	↓Chondrocyte proliferation and migration ↑ Chondrocyte apoptosis	^222^
FLSs	MiR-424	Th17 cells Tregs	FOXP3	↑Th17 cell differentiation ↓ Treg differentiation	^223^
MSCs	MiR-320a	FLSs	CXCL9	↓Activation, migration, and invasion of RA-FLSs	^224^
MSCs	MiR-150-5p	FLSs HUVECs	MMP14, VEGF	↓Synovial hyperplasia ↓ Angiogenesis	^225^
MSCs	MiR-124a	FLSs	ND	↓Proliferation and migration of RA-FLSs ↑ Apoptosis of RA-FLSs	^226^
MSCs	MiR-140-3p	FLSs Chondrocytes	SGK1	↓Proliferation and migration of RA-FLSs ↑ Apoptosis of RA-FLSs ↓ Chondrocyte apoptosis	^227^
MSCs	MiR-146a MiR-155	Tregs	FOXP3, TGF-β, IL-10, RORγt, IL-17, IL-6	↑ Proportion of Tregs	^228^
T cells	MiR-204-5p	FLSs	CRKL, ANGPT1, TGFβR1, TGFβR2	↓ Activation, proliferation, invasion, and inflammatory cytokine production of RA-FLSs	^229^
Th17 cells	MiR-132	Osteoclasts	COX2	↑Osteoclastogenesis	^188^

*miRNA* microRNA, *RA* rheumatoid arthritis, *FLS* fibroblast-like synoviocyte, *MSC* mesenchymal stem cell, *ND* not determined

### FLS-derived exosomal miRNAs

Synovium-derived miRNAs not only orchestrate synovial inflammation but also have a regulatory role in bone and cartilage erosion in RA.^198^ MiRNAs secreted from cells of synovial origin potentially tune gene expression within osteoblasts to govern osteogenesis in RA through exosomal transfer. For instance, overexpression of synovium-derived miR-221-3p impedes osteoblast maturation in RA by modulating Dickkopf2 (DKK2) protein levels to affect Wnt signaling activity.^198^ Exosomes secreted from RA-FLSs carrying miR-486-5p were demonstrated to potentiate the proliferation, differentiation, and mineralization of osteoblasts and thus accelerate bone repair and ameliorate CIA symptoms. Mechanistically, direct interplay between miR-486-5p and TOB1 upregulates BMP/Smad signaling.^221^ Moreover, given that RA-FLS-derived exosomes containing miR-106b significantly disrupts the normal cellular processes of chondrocytes by targeting the PDK4 gene, miR-106b suppression has enormous potential in attenuating cartilage injury in CIA and RA progression.^222^

The manipulation of synovium-derived exosomes with certain miRNAs may direct T-cell differentiation. Exosomes secreted from FLSs with forced expression of miR-424 under hypoxic conditions were shown to disrupt the Th17/Treg balance, increasing the proportion of Th17 cells and simultaneously inhibiting Treg differentiation via FOXP3 inhibition.^223^

### MSC-derived exosomal miRNAs

Several exosomal miRNAs have been reported to move from MSCs to FLSs to protect against the pathological processes of RA, including miR-320a, miR-150-5p, miR-124a, and miR-140-3p.^224–227^ The loading of miR-320a into MSC-derived exosomes is highly effective. After exosomes are taken up by RA-FLSs, the high abundance of exosomal miR-320a substantially restricts the invasive properties of RA-FLSs through interaction of miR-320a with CXCL9.^224^ MiR-150-5p expression in FLSs of RA patients was found to be decreased compared with that in FLSs of OA patients. MSC-derived exosomes carrying miR-150-5p were obtained after transfection of MSCs with a plasmid encoding miR-150-5p. Exosomal miR-150-5p negatively regulated MMP14 and VEGF, contributing to suppression of the migration and invasion of RA-FLSs and the decreased tube formation by HUVECs.^225^ Coincubation of exosomes derived from human MSCs with miR-124a overexpression with RA-FLSs resulted in a significant decline in the number and reduced migration of RA-FLSs.^226^ However, the underlying mechanisms and targets of exosomal miR-124a in the modulation of the aggressive behavior of FLSs in RA were not elucidated in this work. Exosomal miR-140-3p from human umbilical cord MSCs has recently been proven to inhibit RA-FLS growth via SGK1 silencing. Administration of MSC-derived exosomes containing miR-140-3p to a CIA rat model decreased chondrocyte apoptosis and the progression of joint injury.^227^

MSC-derived exosomes also play an immunomodulatory role in RA pathogenesis. Exosomes derived from miR-146a/miR155-transduced MSCs significantly expand Treg populations, which may ultimately lead to the restoration of appropriate T-cell responses in the chronic inflammatory microenvironment of RA.^228^

### T-cell-derived exosomal miRNAs

T-cell-derived exosomes appear to promote the intercellular transfer of miR-204-5p and miR-132. Wu et al. identified human T cells and FLSs as parental and recipient cells of RA-associated exosomal miR-204-5p, respectively. The delivery of miR-204-5p to RA-FLSs repressed cell activation, proliferation, and invasion as well as inflammatory cytokine production.^229^ MiR-132 was found to be packaged into exosomes produced by Th17 cells and released into osteoclast precursors to promote their differentiation into mature osteoclasts via COX2 downregulation. Interestingly, cigarette smoke (CS) can act as an important agonist for AHR, and AHR signaling can result in further forced expression of miR-132 in patients with RA. Increased osteoclastogenesis in arthritic mice with or without CS exposure was blocked by local knockdown of miR-132 in joints.^188^ Unfortunately, despite the high miR-132 expression induced by AHR activation in RA Th17 cells, the authors failed to reveal an intrinsic role of miR-132 in Th17 cell differentiation. In addition to its effect on osteoclastogenesis, Th-17-derived exosomal miR-132 is likely to have promising, albeit unproven, applications for the regulation of arthritis in vivo.

## MiRNA-based diagnostic strategies for RA

Early diagnosis and treatment of RA are the key stopping the progression of the disease and reducing the rate of disability. Owing to the insufficient sensitivity and specificity of the early predictive indicators such as RF and ACPA, the diagnosis mainly depends on the integrated consideration of medical history, clinical manifestations, physical examination results, and radiological characteristics. In many cases, by the time a patient is conclusively diagnosed with RA, irreversible joint damage has already occurred, and the optimal time for treatment has been missed. Targeting characteristic miRNA expression profiles in RA may provide breakthroughs for early diagnosis and therapy.

As mentioned above, miRNAs that are formed and function inside cells can be released into circulating body fluids. Whether these miRNAs are retained or released depends on cell-type-specific miRNA sorting motifs.^66^ Circulating miRNAs of distinct cellular sources are encapsulated in EVs such as exosomes and microvesicles and transferred between cells, thereby establishing intercellular communication.^60,66^ Likewise, some proteins, such as those in Ago2 complexes and lipoproteins, may serve as vesicle-free carriers that transfer extracellular miRNAs to recipient cells.^230,231^ Because they are contained within these carriers as cargo, circulating miRNAs are resistant to degradation by endogenous RNases and thus present in body fluids in a stable form, making it relatively simple to noninvasively extract samples for detection.^232^ Moreover, miRNA expression can be easily detected and accurately quantified by current available techniques, including quantitative reverse transcriptase polymerase chain reaction (qRT‒PCR), in situ hybridization, microarray, and small RNA sequencing. Accordingly, circulating miRNAs are attractive and promising biomarkers for clinical applications in RA, such as aiding early diagnosis, monitoring of the disease course, and prediction of treatment response.

### Contribution to prevention and precise diagnosis

Differential expression profiles of serum miRNAs in RA patients compared to healthy individuals were identified through bioinformatics methods in a multicenter study by Luque-Tévar et al. Among the 223 deregulated serum miRNAs that were assessed for enriched biological functions, the authors further identified a set of miRNAs (miR-106a-5p, miR-143-5p, miR-148b-3p, miR-199a-5p, and miR-346) that seem to target critical genes implicated in RA pathogenesis. Serum levels of miR-106a-5p, miR-148b-3p and miR-199a-5p were decreased whereas serum miR-143-5p and miR-346 levels were increased in patients with RA compared to healthy individuals.^233^ In addition, patients with established rather than early RA exhibited higher expression of serum miR-223 than unaffected controls.^234,235^ A study by Ouboussad et al. suggested a pronounced increase in the expression levels of serum miR-22 after progression from the preclinical state to early RA. Serum miR-22 is therefore deemed an important predictor of the onset and development of RA.^236^ Utilizing the FirePlex multiplex assay, Cunningham et al. identified miR-24-3p, miR-126-3p, miR-130a-3p, miR-221-3p, miR-431-3p, and let-7d-5p as potential biomarkers for RA in a fixed panel of miRNAs linked to immune cell dysfunction. The differential expression of these 6 miRNAs in serum distinguishes RA patients and ACPA-positive at-risk individuals afflicted with arthralgia from healthy subjects.^237^ In addition, circulating miR-103a-3p has been identified as a robust biomarker that can be used to identify populations at risk of imminent RA for timely intervention. Whole-blood miR-103a-3p levels in ACPA-positive, asymptomatic, first-degree relatives were comparable to those in RA patients but increased ~7.6-fold compared with those in healthy subjects.^238^

The levels of plasma miR-22-3p, miR-23b, miR-24-3p, miR-26a-5p, miR-125a-5p, miR-146a-5p, miR-155, and let-7a-5p were elevated in patients with RA in comparison with healthy controls.^239–242^ Among these, the combination of miR-24-3p, miR-26a-5p, and miR-125a-5p showed the greatest diagnostic power for RA.^241^ The upregulation of miR-155 in the plasma of patients with RA seems to agree with the widely reported high expression of miR-155 in diverse RA-associated cells. Unfortunately, another research group revealed lower plasma miR-155 concentrations in RA patients than in healthy subjects.^243^ Indeed, due to the relatively low plasma miR-155 levels,^241^ findings regarding changes in the level of miR-155 in the context of RA have a lower likelihood of being reproduced across studies. MiR-155 expression in serum samples has been shown to be higher in patients with RA than in unaffected controls in at least two independent investigations.^235,244^ Taha et al. later proposed the conflicting view that serum miR-155 expression does not differ between RA patients and controls.^234^ Overall, the intrinsic and extrinsic factors affecting circulating miRNA levels have not been fully characterized. The sex, age, race, and lifestyle of the subjects, techniques employed for miRNA analysis, study design and stage of disease may all contribute to the differences in circulating miRNA signatures.

Circulating exosomal miRNAs have emerged as attractive candidate markers for RA diagnosis. Wu et al. performed global miRNA profiling of plasma exosomes in patients with RA and identified 14 exosomal miRNAs with disturbed expression. The authors subsequently highlighted a decrease in exosomal miR-204a-5p levels in RA patients in comparison to healthy subjects. This finding was confirmed in both the replication and validation groups, which had larger numbers of independent samples.^229^ Likewise, exosome-derived miR-548a-3p and miR-6089, both of which perturb the proliferation and activation of THP-1 macrophages by targeting TLR4, are decreased in serum samples from patients with RA.^245,246^ Focusing on the unique miRNA profiles in serum exosomes from patients with early-stage untreated RA, Rodriguez-Muguruza et al. developed a biomarker panel comprising serum exo-miR-25-3p, exo-miR-451a, and soluble TNF-like weak inducer of apoptosis (TWEAK), an inflammatory cytokine involved in the pathogenesis of RA, that may exhibit superior diagnostic utility over ACPA positivity.^247^ More reproducible studies with expanded sample sizes and designs optimized to assess panel specificity are needed in the future. Two important confounding factors affecting the utility of exosomal miRNAs as biomarkers for RA are the diverse approaches used to isolate miRNAs from body fluids and the difficulty in identifying their cellular origin. With more precise and standardized techniques for separating miRNA-containing exosomes and methods for recognizing exosomes based on their cellular origin and surface protein patterns, exosomal miRNAs will hopefully be developed into robust diagnostic tools for RA.

The final remaining challenge for miRNAs to be used as diagnostic biomarkers in clinical practice is that accurate diagnosis also requires discrimination of RA from other inflammatory autoimmune disorders. Substantial upregulation of miR-22-3p, miR-24-3p, miR-96-5p, miR-134-5p, miR-140-3p, and miR-627-5p, independent of disease activity and seropositivity, was noted and validated in plasma samples from RA patients compared to heathy controls. These differentially expressed miRNAs, however, failed to distinguish RA from systemic lupus erythematosus (SLE) due to their shared autoimmune properties.^248^ Jin et al. discovered that miR-124, miR-448, and miR-551b can serve as reliable diagnostic markers for RA. The expression of these 3 miRNAs in serum samples from patients with RA, however, was comparable to that in patients with SLE, Sjogren’s syndrome, and ulcerative colitis (UC).^249^ Inspiringly, in the past 5 years, several circulating miRNAs have been proposed as suitable biomarkers for the differential diagnosis of RA from related inflammatory autoimmune diseases. For instance, plasma concentrations of miR-16, miR-19b, miR-23a, miR-27a, miR-92a, and miR-223 were significantly higher in patients with RA than in patients with SLE.^250^ In a recent study, Cheleschi et al. first demonstrated the remarkable ability of serum miR-140 levels to discriminate RA from psoriatic arthritis (PsA), suggesting miR-140 as a specific biomarker to distinguish RA from PsA.^251^

Furthermore, it is of practical value to discuss the ability of cell-free miRNAs to differentiate RA from OA. Reduced miR-132 expression was observed in the plasma of patients with RA and patients with OA; however, the change in miR-132 levels was not sufficient to distinguish these two populations.^252^ When compared with OA patients, RA patients demonstrated considerably higher serum miR-125b levels, whereas serum miR-150-5p expression was dramatically diminished.^225,253^ Intriguingly, extracellular miRNAs are also stably present in the synovial fluid of patients with arthritis. Synovial fluid miRNAs, primarily derived from synovial tissue and infiltrating cells within the articular cavity, possess distinct expression patterns from peripheral blood miRNAs.^252^ In patients with RA, the expression of miR-16, miR-132, miR-146a, and miR-223 in synovial fluid was reduced compared to that in plasma,^252^ whereas synovial fluid miR-146b concentrations were increased compared to those in serum.^254^ Synovial fluid levels of miR-16, miR-146a, miR-155, miR-221-3p, and miR-223 were found to be upregulated in RA patients compared with OA patients.^135,252^

### Disease activity and prognosis monitoring

Cell-free miRNAs have been suggested to be promising markers of disease activity, given their marked correlation with a number of clinical parameters in RA. Serum miR-155 expression and serum miR-210 expression in RA were upregulated and downregulated, respectively, and these changes were associated with an increase in TNF-α and IL-1β levels.^244^ A meta-analysis conducted by Bae et al. indicated that forced expression of miR-146a in synovial fluid may result in high erythrocyte sedimentation rates (ESR).^255^ In patients with RA, sustained plasma miR-23b expression suggested active disease and was accompanied by elevated DAS28, ESR, and C-reactive protein (CRP) levels, regardless of the RF or ACPA level.^242^ The level of circulating miR-16 in RA was proven to be significantly negatively correlated with the Disease Activity Score for 28 joints (DAS28).^252^ Notably, the baseline levels of serum miR-16 in patients with early RA, as well as the variations in serum miR-16 levels within the first 3 months following initiation of treatment, appear to have potential in the prediction of disease activity and outcome.^235^ Ciechomska et al. identified circulating miR-146b as a reliable marker of disease activity with improved specificity. The greater the increase in the concentration of serum miR-146b was, the lower the DAS28 based on CRP (DAS28-CRP) and ESR (DAS28-ESR).^254^ Moreover, an inverse correlation between the levels of circulating exosomal miR-204-5p and ESR, CRP, and RF but not DAS28 and ACPA was noted recently.^229^

Patients with a prolonged course of RA are prone to suffer from extra-articular comorbidities, contributing to a bleak prognosis. These comorbidities modify miRNA expression profiles in individuals with RA and are one of the challenges in miRNA biomarker development. The differential expression of serum miR-9-5p in RA patients with peripheral neuropathy suggests that serum miR-9-5p expression can be used as a predictor of RA-associated peripheral neuropathy.^256^ Compared to those in RA patients without lung involvement, the plasma miR-7-5p and miR-214-5p levels were found to be upregulated in RA patients with interstitial lung disease.^257^ Using NGS and random forest analysis, Ormseth et al. identified 8 candidate plasma miRNAs that could be used to monitor the progression of coronary artery calcification in patients with RA, which may support the early identification and management of coronary atherosclerosis.^258^ Furthermore, the serum exosomal miR-155 level is high in RA patients with chronic hepatitis C virus (HCV) infection compared with those without infection.^259^

### Therapeutic response prediction

That miRNAs can be used to predict response to medication offers the opportunity to optimize individualized protocols for more RA patients, which can increase the rate of remission and/or decrease disease activity by allowing patients to switch to alternative therapies. In serum or PBMCs from patients with RA after 3 months of conventional disease-modifying anti-rheumatic drug (cDMARD) treatment, higher baseline levels of miR-15a, miR-16, miR-125b, and miR-223 are associated with a better therapeutic response (DAS28 < 3.2).^110,235,260^ In contrast, high pretherapy concentrations of miR-132-3p, miR-146a-5p, and miR-155-5p in whole blood of RA patients are indicators of inadequate response (DAS28 ≥ 3.2) to MTX therapy.^261^

Biological DMARDs, as the preferred alternative treatment for patients who fail cDMARD therapy, have been confirmed to modify the circulating miRNA expression profile in RA.^233^ In patients with RA who responded well to TNF inhibitor (TNFi)/cDMARD combination treatment, serum miR-23a-3p and miR-223-3p expression was decreased at baseline and significantly increased after 3 months of treatment.^262^ The circulating miR-22, miR-27-3p and miR-886-3p concentrations in patients with RA prior to the initiation of adalimumab/MTX combination therapy are pivotal predictors of a good clinical response. Specifically, RA patients with high plasma miR-27a-3p expression or a combination of decreased serum miR-22 levels and increased miR-886-3p levels are more likely to achieve clinical remission.^263,264^ Notably, adalimumab/MTX combination therapy has been demonstrated to suppress miR-27a-3p expression in the first 3 months after initiation, whereas in nonresponders, an increase rather than a decrease in miR-27a-3p levels has been observed. In addition, reduced miR-5196 expression and elevated circulating miR-146 levels are considered potential markers of good response to TNFi therapy.^262,265,266^

Treatment-naïve patients with RA who have high serum miR-125b expression levels tend to have disease that can be adequately controlled by rituximab therapy.^253^ Importantly, considering that increased HCV activity is associated with rituximab response, serum exosomal miR-155, as a suppressor of HCV replication in hepatocytes, may be applied to monitor the HCV load in RA patients during the duration of rituximab therapy.^259^

Serum miR-432-5p concentrations were decreased in patients with RA in remission but surprisingly upregulated in nonremission patients and patients who relapsed following 5 years of tofacitinib therapy in a trial.^267^ Nasonov et al. recently published the first randomized controlled phase III trial of olokizumab (OKZ), a monoclonal antibody targeting IL-6, in RA, in which they evaluated the efficacy and safety of OKZ in RA patients who failed MTX monotherapy.^268^ Furthermore, baseline plasma levels of miR-26b, miR-29, miR-451, and miR-522 in RA patients appear to be predictors of response to OKZ therapy, although the findings were derived from a Russian population.^269^

## MiRNA-based therapeutic strategies for RA

Modern therapies for RA aim to achieve remission or at least low disease activity within 6 months, which is fulfilled by a treat-to-target strategy that involves rigorous disease control and individualized management.^270^ To date, the first-line medication for RA patients has been cDMARDs, primarily MTX, supplemented with glucocorticoids and/or nonsteroidal anti-inflammatory drugs. The emergence of novel DMARDs in the past 2 decades, including small molecule inhibitors and biologic agents, has provided additional opportunities for improving the course and prognosis of this disease.^7,14^ Detailed classifications and descriptions of modern pharmacologic therapies for RA and key cells and receptors/pathways targeted by these strategies have been thoroughly reviewed elsewhere.^7,271^ Nevertheless, current treatment strategies sometimes produce only partial responses accompanied by inevitable side effects or require long-term use due to the high likelihood of relapse following therapy discontinuation.^270^ Considering the challenge of re-establishing immune homeostasis and remodeling after joint destruction, achieving sustained remission or retaining low disease activity remains elusive. Therefore, the continued development of safe and efficacious new RA therapeutic agents with the aim of preventing disease progression and improving patient quality of life is warranted.

### Modulation of miRNAs by modern RA pharmacologic therapies

As RA is a multifactorial and stage-specific inflammatory disease, the onset, progression, and persistence of RA inevitably require the involvement of a range of cells and cytokines.^271^ Focusing on the effects of modern pharmacologic therapies on miRNAs in RA will help further elucidate definitive molecular or cellular nodes in complex miRNA regulatory networks that subserve disease and aid the development of novel therapeutic strategies.

MTX is widely used as an anchor drug for RA treatment. Intriguingly, administration of MTX alters the global expression profile of miRNAs in RA-FLSs, resulting in sustained expression of miR-877-3p. Dysregulation of miR-877-3p expression limited the growth and migration of RA-FLSs by significantly reducing GM-CSF and CCL3 transcript levels.^272^

Upregulation of miR-29b in RA may be associated with TNF-α induction. Repressed miR-29 expression in RA patients treated with infliximab contributes to enhanced resistance of PBMCs to apoptosis by inactivating HBP1 signaling.^273^ Administration of the TNFi adalimumab to patients with RA downregulated miR-155 levels in macrophages and consequently reversed the defect in M2 polarization.^115^ Low global miRNA levels in RA neutrophils were observed, which may have been mediated by the release of excessive levels of inflammatory cytokines, such as TNF-α and IL-6, and/or a defect in miRNA processing as a result of DICER inadequacy. Treatment with the anti-TNF agent infliximab and the anti-IL-6 agent tocilizumab dramatically reversed the global downregulation of miRNAs in RA neutrophils, improved miRNA processing and decreased the production of various cytokines and chemokines, which blocked the activation of RA neutrophils.^177^ Moreover, tocilizumab was found to upregulate serum miR-146a-5p levels in treated RA patients. Conversely, treatment of an in vitro coculture system including monocytes and fibroblasts with tocilizumab downregulated miR-146a-5p expression.^274^ One explanation is that the serum may reflect the state of synovial joints, where interactions between multiple cell types may produce different results than those derived from in vitro cell lines. Despite these inconsistencies, tocilizumab treatment has been demonstrated to regulate miR-146a-5p expression, which is partially involved in the restriction of angiogenic potential in RA patients.^274^

Abatacept is a T-cell costimulation modulator consisting of an extracellular domain of human cytotoxic T-lymphocyte-associated antigen 4 (CTLA-4) and a modified Fc portion of human IgG1.^271^ According to Hayakawa et al., plasma miR-766-3p levels were lower in abatacept-treated RA patients than in untreated patients. The authors focused on not only the possibility of applying plasma miR-766-3p as a biomarker for RA but also its function in different cell lines in suppressing the inflammatory response through indirect NF-κB signaling inactivation.^275^

### MiRNA-based gene therapies in RA

Overall findings regarding the involvement of miRNAs in mediating pathogenic processes in RA suggest enormous potential for their therapeutic manipulation. Although a considerable number of miRNAs differentially expressed in RA have been discovered over the years, and some corresponding preclinical studies have been conducted, the development of miRNA therapeutics is in its infancy. It is still difficult to translate these preliminary investigations into clinical practice for RA therapy. Bare miRNAs in body fluids are bound to be rapidly degraded by RNases, which prevents them from reaching the target sites to perform functions. In addition to having poor biostability, due to their large molecular size and strong anionic charge, miRNAs are unable to pass through the plasma membrane, and therapeutic miRNAs are prone to off-target effects.^276^ A specific miRNA may exert diverse effects in RA pathogenesis, depending on the cell type in which it is expressed. Certain miRNAs are protective against RA, regardless of their expression in clinical samples. Hence, there are two main challenges in developing miRNA-based therapies for RA: (i) developing a proper and comprehensive understanding of miRNA function, which will facilitate the selection of miRNA candidates with maximum target efficacy and knowledge of whether to inhibit or increase their endogenous expression; and (ii) rationally designing optimal miRNA delivery vehicles that allow tissue- or cell-specific targeting, which will lead to outstanding biostability and cellular uptake as well as minimal toxicity and low probability of off-target effects of miRNA candidates.

#### MiRNA inhibition and replacement therapy

The central action of miRNAs is to restrain gene expression by binding and silencing specific mRNAs, which in turn modulates cellular function. Accordingly, miRNA-based gene therapies aim to artificially interfere with target miRNA activity in RA, managing RA-related gene expression and protein synthesis via the silencing of overexpressed pathogenic miRNAs and increasing the expression of underexpressed beneficial miRNAs.

Currently, clinical studies of miRNA-based therapeutic strategies mainly use inhibitors and mimics of miRNAs. MiRNA inhibitors are synthetic single-stranded RNA molecules consisting of nt complementary to endogenous miRNAs, whereas miRNA mimics are synthetic double-stranded RNAs that bypass the synthesis process of endogenous mature double-stranded miRNAs and achieve high expression of specific miRNAs in a short duration. Synthetic double-stranded miRNAs and antisense oligonucleotides are frequently used for in vitro functional assays of miRNAs, but their chemical modification is required to increase affinity, biostability and tissue and cellular uptake when they are applied in vivo to modulate deregulated miRNAs.^277^ In most cases, specifically chemically modified miRNA agomirs and antagomirs are recommended for use in validation in in vivo animal experiments. Injection of chemically modified miR-708-5p mimics into the tail vein of rats with CIA ameliorated arthritis scores and ankle histopathology by blocking Wnt3a/β-catenin signaling activation.^278^ In contrast, intravenous administration of an miR-145-5p agomir resulted in reduced OPG expression in ankle joints and a consequent increase in the severity of joint structural destruction in mice with CIA.^192^ Moreover, CIA mice injected intravenously with an miR-34a antagomir exhibited improved arthritis phenotypes and symptoms, accompanied by a restored Treg proportion.^165^

Systemic administration of miRNAs tends to result in unsatisfactory biodistribution and short-term serum miRNA levels owing to preferential accumulation in the liver and rapid renal clearance.^277^ Thus, local administration seems to be more appropriate for delivering miRNAs in RA to achieve sustained therapeutic concentrations in the inflamed joints, avoiding repeated injections or a single administration of high doses. AIA mice injected intra-articularly with an miR-132 antagomir demonstrated a significant reduction in the number of periarticular osteoclasts.^188^ Consistent with the in vitro identification of the miR-141-3p/FoxC1/β-catenin axis regulating the pathological behaviors of RA-FLSs, manipulation of miR-141-3p overexpression in the joint cavity suppressed the expression of FoxC1 and β-catenin in the CIA rat model. Synovial hyperplasia, inflammation, and cartilage degradation, as expected, were inhibited in CIA rats treated with miR-141-3p agomir.^99^ Following injection of pre-miR-124 into a single ankle joint of rats with AIA, apart from accumulation at the injection site, mature miR-124 was also distributed to distant joints, leading to alleviated arthritic tissue destruction in multiple joints.^190^

Another approach of miRNA replacement and inhibition therapy in RA is to use lentiviruses as vectors for in vivo gene transfer. Lentivirus vector systems are ideally suited to mediate miRNA interference in RA due to their wide range of transduced tissues and efficient delivery of miRNAs. Wu et al. demonstrated that intra-articular administration of an miR-204-5p-overexpressing lentivirus could dramatically mitigate the development of CIA.^229^ Intra-articular injection of a pre-miR-140-containing lentivirus ameliorated synovial inflammation and pannus formation, which was in part due to the high abundance of FLSs mediated by miR-140 in the synovium of arthritis mice.^279^ In one of our previous studies, we delivered miR-106b inhibitors into mice with established CIA via orbital injection of lentiviral vectors to neutralize endogenous miR-106b expression in inflamed joints. Lentivirus-mediated miR-106b diminution decreased the ratio of RANKL to OPG and interfered with inflammatory cytokine production at the local and systemic levels.^183^ Building on the recognition of miR-106b as a putative target for RA therapy, other researchers have further concluded that miR-106b inhibition in the CIA mouse model contributes to the rebalancing of the RANKL/RANK/OPG system and restoration of cartilage degradation, which is the result of PDK4 upregulation.^222^ However, some concerns with lentivirus vector-mediated miRNA interference have also been uncovered, including off-target effects, innate immune responses, and alterations in endogenous miRNA pathways. Future research efforts will focus on finding more feasible solutions to mitigate these potential risks and enable the clinical application of this technology.

#### MSC-derived exosomes as carriers for miRNAs

The endogenous properties of exosomes enable them to be natural delivery vehicles for miRNAs and improve cell-free therapeutic strategies for RA.^280,281^ Among the various cell-derived exosomes, those of MSC origin exert excellent immunomodulatory effects and thus are likely to be the most ideal agents for the delivery of therapeutic miRNAs in autoimmune diseases.

CIA rats treated with bone marrow-derived mesenchymal stem cell (BMSC)-derived exosomes carrying miR-192-5p showed a delayed inflammatory response and a decrease in osteoclast numbers.^282^ Exosomal miR-21 secreted from BMSCs was found to be incorporated into mouse FLSs, inhibiting inflammatory cytokine generation via the miR-21/TET1/KLF4 axis. BMSC-derived exosomal miR-223 was also found to exert anti-inflammatory activity in macrophages by restraining activation of the NLRP3 inflammasome. Administration of exosomal miR-21 in mice with CIA apparently attenuated inflammatory infiltration in synovial tissues, whereas the in vivo efficacy of exosomal miR-223 has not been discussed.^283,284^ It has been proven that MSC-secreted exosomes can transfer miR-320a and miR-150 into RA-FLSs, thereby altering their pathological properties. Tail vein injection of MSC-derived exosomes containing miR-320a into CIA mice alleviated arthritis severity, which was correlated with suppressed CXCL9 levels.^224^ MSC-derived exosomal miR-150 interfered with the migration, invasion, and tube formation of RA-FLSs through a mechanism involving MMP14 and VEGF. Thus, intraperitoneal administration of exosomal miR-150 in mice with CIA led to the amelioration of synovial hyperplasia and angiogenesis.^225^ Recently, Huang et al. reported that injection of miR-140-3p packaged in human umbilical cord mesenchymal stem cell-derived exosomes into CIA rats had therapeutic effects, including reducing the inflammatory response, oxidative stress, synoviocyte proliferation and cartilage damage.^227^

Notably, exosomal surfaces can be finely edited by bioengineering to augment their stability, bioactivity and efficiency in targeting specific cells and tissues. Based on metabolic glycoengineering-mediated click chemistry, You et al. developed dextran sulfate-decorated exosomes (DS-EXOs) secreted from surface-modified adipose-derived stem cells to target macrophages in RA. Let-7b-5p and miR-24-3p encapsulated in the engineered DS-EXOs were internalized by activated macrophages and drove M1 macrophages to polarize toward the M2 phenotype. Intravenous injection of DS-EXOs into CIA mice at a lower dose than native exosomes resulted in increased accumulation in the inflamed joints and consequent relief of arthritis symptoms, ultimately decelerating CIA progression.^285^

Despite these preclinical studies showing the therapeutic potential of MSC-derived exosomes in RA, there are still some gaps regarding safety and efficacy that limit their clinical application.^286^ The underlying transport and cellular mechanisms that underly the observed clinical benefits of exosomal miRNAs require in-depth characterization. The current technologies of exosome isolation and purification are relatively immature. Furthermore, the optimal administration regimen for exosomes, including route and dose, is unknown. The poor in vivo biodistribution of MSC-derived exosomes after systemic administration, which affects the efficacy of practical applications, needs to be addressed.

#### Synthetic materials as miRNA delivery vehicles

Although liposomes are widely used as in vitro transfection reagents due to their low immunogenicity, their in vivo application is often restricted by toxicity linked to compound composition and structure.^287^ Encouragingly, several cationic lipid-based vectors have been successfully utilized to improve the in vivo delivery of therapeutic miRNAs in murine models of RA.

Injection of lipoplexes encapsulating miR-17 mimics into the ankle joints protected CIA mice from synovitis, cartilage degradation and bone erosion. Forced miR-17 expression markedly inhibited pro-inflammatory cytokine (for example, IL-6) secretion, synovial immune cell infiltration, and osteoclast formation by blocking JAK1/STAT3 signaling.^93^ Sujitha et al. employed poly-ethylene glycol (PEG)-grafted polyethylenimine (PEI) to deliver miR-23a, a functional miRNA associated with Wnt1/β-catenin signaling modulation. MiR-23a incorporated within PEGylated PEI polyplexes was systemically administered to AIA rats, in which it achieved multifaceted benefits in terms of inducing disease remission.^288^ A previous study validated the role of miR-124 in impeding osteoclast differentiation via direct interaction with NFATc1 mRNA.^190^ To enable MTX and miR-124 combination therapy, methotrexate-conjugated polymer hybrid micelles (M-PHMs) with efficient cellular uptake, optimized transfection efficiency and decreased cytotoxicity were developed. M-PHMs were coloaded with miR-124 to target activated macrophages in inflamed joints. The application of M-PHM/miR-124 complexes led to significant alleviation of arthritis symptoms through anti-inflammatory and anti-bone erosion effects in the AIA rat model.^289^

Of note, lipoplexes containing miR-146a mimics formulated with DMAPAP/DOPE cationic liposomes efficiently delivered miR-146a to Ly6C^high^ monocytes and diminished inflammation-induced local bone loss; however, these effects failed to ameliorate the overwhelming inflammation in both CIA and STA mouse models.^129^ Likewise, CIA mice injected intravenously with double-stranded miR-146a/atelocollagen complexes were resistant to bone erosion but not synovitis.^123^ These results are consistent with the overexpression of miR-146a noted in various cells and tissues of RA, and miR-146a seems to lose its normal function as a sensor to orchestrate the inflammatory response.

MiRNA-loaded nanoparticles (NPs) are another well-tested carrier for miRNAs. Poly(lactide-co-glycolide) (PLGA) NPs and poly (cyclohexane-1,4-diylacetone dimethylene ketal) (PCADK) NPs have been applied to codeliver miR-124 and ketoprofen into rats with AIA. When administered systemically, miR-124 and ketoprofen encapsulated in these two delivery systems effectively decelerated the progress of AIA. Compared to PLGA NPs, acid-responsive PCADK NPs achieved accelerated release of payloads in response to the slightly acidic microenvironment of inflamed joints, suggesting superior therapeutic efficacy.^290,291^

Based on evidence indicating overexpression of miR-30-5p in synovial tissue samples from patients with RA, Zhang et al. fabricated cell membrane-penetrating peptide (CADY)-modified mesoporous silica nanoparticle (MSN)/miR-30-5p inhibitor complexes for targeted delivery into RA-FLSs. The reported systemic delivery strategy using these miR-30-5p inhibitor-containing complexes in coordination with a near-infrared response MSN-based vehicle that governs triptolide (TP) release is promising for RA treatment.^292^

Moreover, after repeated systemic administration in zymosan A-induced arthritis (ZIA) mice, passive accumulation of the formulated nanocomplexes (NCs) loaded with miR-21 and IL-4 in inflamed joints was observed owing to their prolonged serum half-life (t_1/2_). MiR-21 cooperates with IL-4 to orchestrate the composition of the osteoimmune microenvironment in ZIA, eliciting pronounced anti-inflammatory and tissue repair outcomes via NF-κB signaling inactivation and M2 polarization promotion. Intriguingly, the NC-mediated codelivery of miR-21 and IL-4 to different target sites was carried out in a hierarchical manner under spatiotemporal control. Specifically, instructed by the micro-acidic inflammatory surroundings, the poly-l-lysine-*cis*-aconitic anhydride (PLL-CA) in the outer layer of the NCs undergoes charge reversal. The switch of PLL-CA from negative to positive first leads to the liberation of surface-adsorbed IL-4 to the extracellular compartments of the inflamed synovium, followed by exposure of the cationic membrane-penetrating, helical polypeptide (PG)/miR-21 inner core, which allows intracellular transport into macrophages.^293^

## Conclusions and perspectives

The recognition of miRNAs as fine-tuners of gene expression and the abundance of intensive relevant research have updated our fundamental understanding of RA pathogenesis. A plethora of deregulated miRNAs are implicated in regulating the differentiation, development, homeostasis and function of multiple cell types in different tissues and developmental stages, thereby contributing to tissue inflammation and damage in RA. The miRNA-mediated crosstalk between diverse cell lines in RA also represents an important part of the disease process. Notably, the complicated interactions and integration of multilayer signaling between miRNAs and other epigenetic factors are indispensable for RA pathogenesis but remain largely unknown. Many mechanistic studies concerning miRNA–mRNA interactions have identified a growing list of miRNA target genes in FLSs, immune cells and bone-related cells, allowing a glimpse of the potential functional scope of these miRNAs in inflammatory cascades, the immune response and bone homeostasis. Nevertheless, there is currently a lack of sufficient in vivo experimental evidence to address the pleiotropic role of miRNAs in RA disease states. More studies should be conducted in animal models of inducible arthritis, preferably utilizing conditional gene knockout/knock-in strategies, to further deduce the function of specific miRNAs in distinct cell subsets of RA. Furthermore, the initiation, development and persistence of RA rely on the synergistic actions of miRNAs, which constitute a complex network of intracellular signaling modulation. Thus, although the capacity of a single miRNA to target a broad range of genes simultaneously is exciting and being extensively researched, the combinatorial effects of global miRNAs on gene expression as well as pathological phenotype in RA warrant further exploration.

Accurate early diagnosis and feasible new drug therapies that facilitate the achievement of remission are urgently needed for patients with RA. Circulating miRNAs have been proposed as novel biomarkers for RA. Given that the field is still in its infancy, more large-scale cohort studies are required to verify the superiority and clinical utility of miRNAs as diagnostic, predictive and prognostic markers in RA.

In addition, a profound understanding of miRNA deregulation contributes to the clinical development of miRNA-based therapies for RA. The ability of miRNAs to target multiple genes and pathways renders them promising therapeutic targets. Unfortunately, this feature has also conferred significant risk of off-target effects and unpredictable side effects owing to the systemic delivery of a particular miRNA in the majority of preclinical studies. In some cases, simultaneous regulation of the expression and function of multiple miRNAs might lead to better clinical outcomes in RA patients. However, the widespread effects of miRNAs on several targets in different tissues is potentially capable of shutting down entire pathways. The remaining questions regarding miRNA tissue distribution and systemic toxicity imply that before using miRNAs in clinical practice, substantial work is still required to comprehensively appraise the therapeutic potential of miRNAs and to obtain optimized protocols. The design of safe and effective delivery systems that enable miRNA aggregation in inflamed joints and specific target cell types in RA remains the key to bringing miRNAs into the clinic. MiRNA-mediated gene regulation is highly sensitive to the cell type and context. Strategies targeting monocytes and MSC-based strategies appear to be ideal therapeutic approaches since these cells serve as the main source of pathogenic cells in the development of RA. Exosomal miRNAs driving intercellular communication have shown substantial potential as candidates for RA treatment. In addition to linking miRNAs to RA therapy from a biological perspective, encouraging results have been achieved using nanomaterial-based engineering strategies for the local delivery of miRNAs into joint tissues.

In conclusion, comprehensive insights into the pathogenesis of RA gained from a multidisciplinary approach will continue to accelerate the clinical utilization of miRNAs as novel biomarkers and therapeutic targets, thus moving toward a new era of individualized management for patients with RA.
